# Syntheses, Raman spectroscopy and crystal structures of alkali hexa­fluorido­rhenates(IV) revisited

**DOI:** 10.1107/S2056989018005297

**Published:** 2018-04-06

**Authors:** James Louis-Jean, Samundeeswari Mariappan Balasekaran, Dean Smith, Ashkan Salamat, Chien Thang Pham, Frederic Poineau

**Affiliations:** a Department of Chemistry, University of Nevada Las Vegas, 4505 South Maryland Parkway, Las Vegas, Nevada, 89154, United States; b Department of Physics and Astronomy and *HiPSEC*, University of Nevada Las Vegas, 4505 South Maryland Parkway, Las Vegas, Nevada, 89154, United States; c Department of Chemistry, Hanoi University of Science, Hanoi, Vietnam

**Keywords:** rhenium, fluorine, crystal structure, Raman spectroscopy, isotypism

## Abstract

The *A*_2_[ReF_6_] (*A* = K, Rb and Cs) salts are isotypic and crystallize in the trigonal space group type *P*  *m*1, adopting the K_2_[GeF_6_] structure type. Common to all *A*_2_[ReF_6_] structures are slightly distorted octa­hedral [ReF_6_]^2−^ anions with an average Re—F bond length of 1.951 (8) Å. In those salts, symmetry lowering on the local [ReF_6_]^2−^ anions from *O_h_* (free anion) to *D*_3*d*_ (solid-state structure) occur. The distortions of the [ReF_6_]^2−^ anions, as observed in their Raman spectra, are correlated to the size of the counter-cations.

## Abstract 2

The *A*_2_[ReF_6_] (*A* = K, Rb, Cs) salts are isotypic and crystallize in the K_2_[GeF_6_] structure type.

## Chemical context   

The hexa­fluorido­rhenate(IV) anion has been known for 80 years but its chemistry is understudied with respect to the heavier halogen analogs (Ruff & Kwasnik, 1934 ▸). The scarcity of [ReF_6_]^2−^ salts is attributed to the difficulties in their preparation and purification. K_2_[ReF_6_] was the first hexa­fluorido­rhenate(IV) salt to be reported; it was prepared from the solid-state melting reaction (SSMR) of K_2_[ReBr_6_] with KHF_2_ (Ruff & Kwasnik, 1934 ▸). Almost two decades later, ten salts comprising the [ReF_6_]^2–^ anion and with different counter-cations (Rb^+^, Cs^+^, PPh_4_^+^ (Ph = C_6_H_5_), [Ni(NH_3_)_6_]^2+^, [Co(NH_3_)_6_]^3+^, {[Co(NH_3_)_6_](NO_3_)}^2+^, {[Cr(NH_3_)_6_](NO_3_)}^2+^, [Co(NH_3_)_5_Cl]^2+^, [Cr(NH_3_)_5_Cl]^2+^, [Co(NH_3_)_4_(CO_3_)]^2+^) had been reported (Peacock, 1956 ▸; Weise, 1956 ▸; Pedersen *et al.*, 2014 ▸; Brauer & Allardt, 1962 ▸). Those salts were prepared by cation metathesis starting from (NH_4_)_2_[ReF_6_] or K_2_[ReF_6_]. However, the synthetic procedure to prepare (NH_4_)_2_[ReF_6_] or K_2_[ReF_6_] was not explained in detail. To date, only the structures of two [ReF_6_]^2−^ salts have been characterized by single crystal X-ray diffraction (SCXRD): K_2_[ReF_6_] (measured at 292 K) and (PPh_4_)_2_[ReF_6_]·H_2_O (measured at 122 K) (Clark & Russell, 1978 ▸; Pedersen *et al.*, 2014 ▸). Similarly, the synthesis of the K_2_[TcF_6_] congener, which was reported in 1963, involves the SSMR of K_2_[TcBr_6_] with KHF_2_ followed by an aqueous work-up (Schwochau & Herr, 1963 ▸). However, [TcF_6_]^2−^ salts have been reinvestigated recently (Balasekaran *et al.*, 2013 ▸), and various routes for the different salts of *A*_2_[TcF_6_] [*A* = Na, K, Rb, Cs and N(CH_3_)_4_] were reported. These salts were characterized by Raman and IR spectroscopy and by SCXRD. The *A*_2_[ReF_6_] salts could serve as suitable precursors to explore the chemistry of rhenium in the oxidation state IV.

Here, we revisited the synthesis of *A*_2_[ReF_6_] (*A* = K, Rb, Cs) salts and report their crystal structures determined from single crystal data, and their Raman spectra.

## Structural commentary   

**Figure 1:**
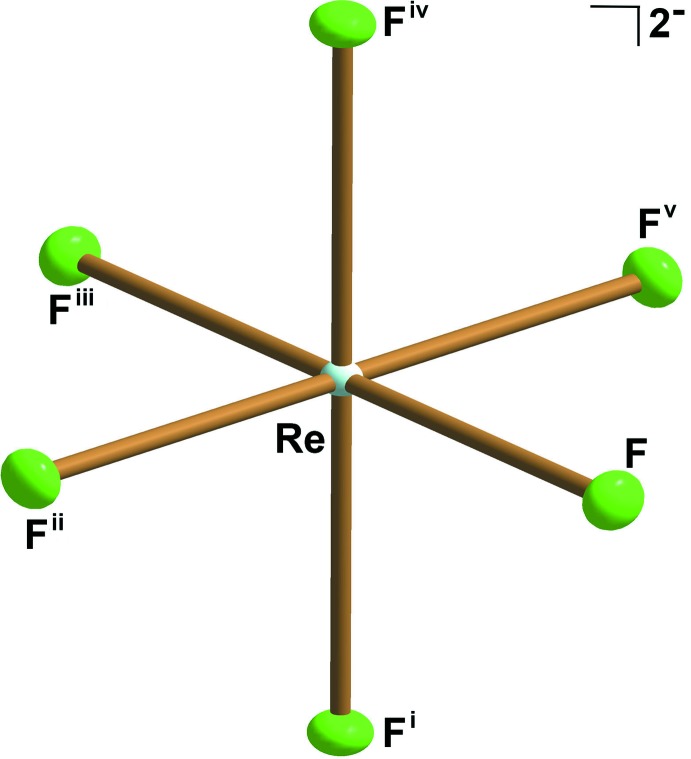
Representation of the [ReF_6_]^2−^ anion in Cs_2_[ReF_6_]. Displacement ellipsoids are drawn at the 50% probability level. [Symmetry codes: (i) −*x*, −*y*, −*z*; (ii) *x* − *y*, *x*, −*z*; (iii) −*x* + *y*, −*x*, *z*; (iv) −*y*, *x* − *y*, *z*; (v) *y*, −*x* + *y*, −*z*.]

The title alkaline metal salts *A*_2_[ReF_6_] (*A* = K, Rb, Cs) are isotypic. They adopt the K_2_[GeF_6_] structure type (Hoard & Vincent, 1939 ▸) and crystallize in the trigonal space group type *P* 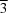 *m*1 (Table 1 ▸), just like the related *A*_2_[TcF_6_] (*A* = K, Rb, Cs) compounds (Balasekaran *et al.*, 2013 ▸). Selected bond lengths and angles of the series of [ReF_6_]^2−^ anions of the present work and the reported [TcF_6_]^2−^ salts (Balasekaran *et al.*, 2013 ▸) are presented in Table 1 ▸. Representative for all other title compounds, the [ReF_6_]^2−^ anion of the Cs_2_[ReF_6_] salt is given in Fig. 1 ▸. The Re^IV^ atom is located on a position with site symmetry 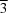 *m.* (Wyckoff position 1*a*) at the origin of the trigonal unit cell. The six symmetry-related fluorine ligands form a slightly distorted octa­hedral coordination sphere around the rhenium(IV) atom. The Re—F bond lengths for the K, Rb, and Cs salts of [ReF_6_]^2−^, 1.948 (3), 1.945 (7) and 1.9594 (18) Å, respectively, are longer than the Tc—F bond lengths for the congener K, Rb, and Cs salts of [TcF_6_]^2−^, 1.928 (1), 1.933 (3), and 1.935 (5) Å, respectively (Balasekaran *et al.*, 2013 ▸).

In *A*_2_[ReF_6_] (*A* = K^+^, Rb^+^, Cs^+^), each cation is located on a position with site symmetry 3*m.* (Wyckoff position 2*d*) and is surrounded by twelve neighboring F atoms resulting in a [3 + 6 + 3] arrangement with three groups of fluoride ligands with distances of 3.0955 (19) Å (three of such), 3.1655 (6) Å (six of such), and 3.224 (2) Å (three of such) for the Cs^+^ salt as a representative of the three [ReF_6_]^2−^ salts. These bond-length distributions are also found in the K^+^ and Rb^+^ salts of the [ReF_6_]^2−^ complexes. This correlates well and confirms that *A*_2_[ReF_6_] salts are isotypic with K_2_[GeF_6_] and the congener *A*_2_[TcF_6_] (*A* = K^+^, Rb^+^, Cs^+^) (Balasekaran *et al.*, 2013 ▸; Hoard & Vincent, 1939 ▸). In comparison with the previous structure determination of K_2_[ReF_6_] (Clark & Russell, 1978 ▸), the current redetermination resulted in better reliability factors, together with a more precise determination of lattice parameters and atomic coordinates.

## Raman spectroscopy   

**Figure 2:**
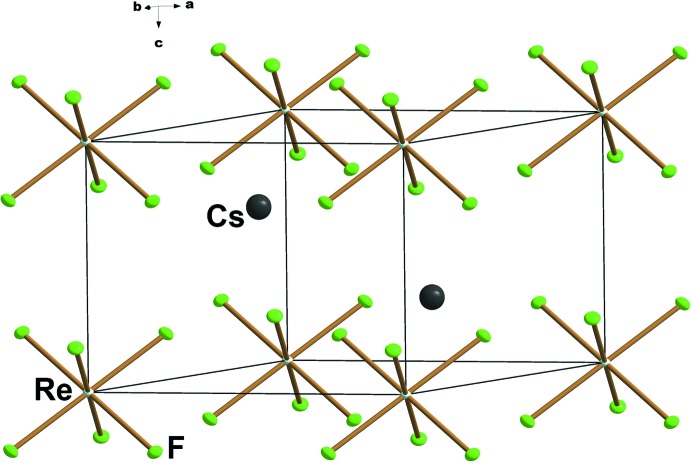
A packing diagram of Cs_2_[ReF_6_]. Displacement ellipsoids are drawn at the 50% probability level.

**Figure 3:**
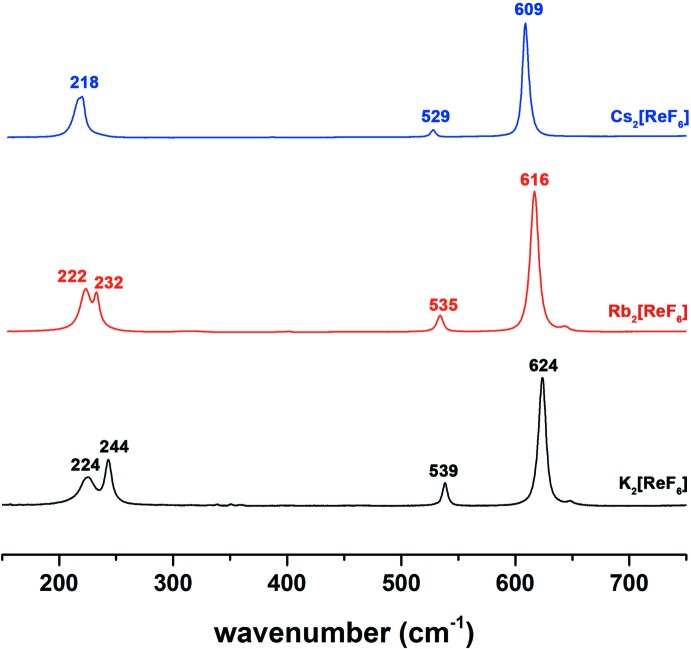
Raman spectra of *A*_2_[ReF_6_] (*A* = K, Rb, Cs).

As reported previously for K_2_[ReF_6_] and *A*_2_[TcF_6_] (*A* = K, Rb, Cs) (Bettinelli *et al.*, 1987 ▸; Balasekaran *et al.*, 2013 ▸), the [ReF_6_]^2−^ anions are compressed along the crystallographic *c* axis, thus lowering the ideal mol­ecular symmetry of the [ReF_6_]^2−^ anions from *O_h_* to *D*_3*d*_ in the solid state. The representive unit-cell plot of Cs_2_[ReF_6_] is given in Fig. 2 ▸. The effect of symmetry lowering among the alkali metal salts of [TcF_6_]^2−^ and its correlation with the vibrational spectra are well described (Balasekaran *et al.*, 2013 ▸). Here, a similar trend occurs for the *A*_2_[ReF_6_] series (*A* = K, Rb, Cs; Fig. 3 ▸). In the case of K_2_[ReF_6_], the Raman spectrum exhibits four bands at 624, 539, 244 and 224 cm^−1^. The latter two vibrations correspond to the *F*_2*g*_ band split due to the symmetry lowering. In the Raman spectra of *A*_2_[*M*F_6_] complexes (*A* = K, Rb, Cs; *M* = Tc, Re), the *F*_2*g*_ splitting decreases from K_2_[ReF_6_] to Cs_2_[ReF_6_] due to differences in *M*—F bond length. Furthermore, the slight increase of *M*—F bond lengths from K_2_[*M*F_6_] to Cs_2_[*M*F_6_] are well represented in the Raman spectra which causes the Raman bands to shift to lower wavenumbers.

## Synthesis and crystallization   

Ammonium perrhenate, ammonium bifluoride, potassium fluoride, rubidium fluoride, cesium fluoride, and hydro­bromic acid (48%) were purchased from Sigma Aldrich and used without any further purification. This work was performed in a well-ventilated fume hood due to the corrosive nature of bifluoride. K_2_[ReBr_6_] was prepared as described in the literature (Watt *et al.*, 1963 ▸), and the detailed synthesis of *A*_2_[ReF_6_] (*A* = K, Rb, Cs) is described below. Single crystals of *A*_2_[ReF_6_] (*A* = K, Rb, Cs) were obtained by slow evaporation at room temperature of an aqueous solution of the respective salt.

**Synthesis of K_2_[ReF_6_]**

K_2_[ReF_6_] was prepared by melting K_2_[ReBr_6_] (2 g, 2.69 mmol) with excess KHF_2_ (14 g, 0.18 mol) in a nickel crucible at 673 K for 30 min in a box furnace. The resulting greyish solid product formed was allowed to cool to room temperature and was washed first with MeOH (4 × 10 ml). Subsequently, the product was washed with several aliquots of an H_2_O/MeOH mixture (3 × 5 ml, 1:4 volume ratios) and centrifuged. The pink solid obtained was dissolved in warm water (5–10 ml, 353 K) and evaporated slowly at room temperature. The resultant pink crystals of K_2_[ReF_6_] were recrystallized from warm water (5 ml, 353 K) and colorless crystals of K_2_[ReF_6_] were obtained. Yield: 661 mg, 1.7 mmol (65%). IR (KBr, cm^−1^): 518, 484 sh (Re—F).

**Syntheses of** ***A*** **_2_[ReF_6_] (** ***A*** **= Rb, Cs) salts**

K_2_[ReF_6_] (151 mg, 0.4 mmol) was dissolved in 4 ml of hot water (353 K). *M*F (*M* = Rb, Cs) (0.8 mmol) dissolved in 1 ml of hot water (353 K) was added dropwise. The solution was allowed to evaporate slowly at room temperature. Crystals of Rb_2_[ReF_6_] and Cs_2_[ReF_6_] were formed in 24 h and washed first with cold water (3 × 2 ml) to remove other fluoride impurities followed by iso­propanol (3 × 1 ml), and diethyl ether (3 × 1 ml). Rb_2_[ReF_6_] yield: 156 mg, 0.33 mmol (83%). IR (KBr, cm^−1^): 521 (Re—F). Cs_2_[ReF_6_] yield: 175 mg, 0.276 mmol (77%). IR (KBr, cm^−1^): 507, 480 sh (Re—F).

IR spectra were measured on a Shimadzu IR Affinity-1 spectrometer between 400 and 4000 cm^−1^. Raman spectra were recorded on a HORIBA T64000 triple spectrometer operating at 30 mW in subtractive mode. The spectra were taken from pure single crystals at room temperature using the 514.5 nm (Kr/Ar) laser line.

## Refinement   

Crystal data, data collection and structure refinement details are summarized in Table 2 ▸.

## Supplementary Material

Crystal structure: contains datablock(s) global, global_1, global_2, SMB_K2ReF6_1, SMB_Rb2ReF6_j, SMB_Cs2ReF6_1a. DOI: 10.1107/S2056989018005297/wm5432sup1.cif

Structure factors: contains datablock(s) SMB_K2ReF6_1. DOI: 10.1107/S2056989018005297/wm5432SMB_K2ReF6_1sup2.hkl

Structure factors: contains datablock(s) SMB_Cs2ReF6_1a. DOI: 10.1107/S2056989018005297/wm5432SMB_Cs2ReF6_1asup3.hkl

Structure factors: contains datablock(s) SMB_Rb2ReF6_j. DOI: 10.1107/S2056989018005297/wm5432SMB_Rb2ReF6_jsup4.hkl

Structure factors: contains datablock(s) SMB_ReF6_f. DOI: 10.1107/S2056989018005297/wm5432SMB_ReF6_fsup5.hkl

Hexafluororhenate(IV) - revisited. DOI: 10.1107/S2056989018005297/wm5432sup6.pdf

Syntheses, Raman spectroscopy and crystal structures of alkali hexafluoridorhenates(IV) - revisited. DOI: 10.1107/S2056989018005297/wm5432sup7.pdf

CCDC references: 1834616, 1834615, 1834614

Additional supporting information:  crystallographic information; 3D view; checkCIF report

## Figures and Tables

**Table 1:** Structural details (Å, °) of the [ReF_6_]^2−^ anion in this study and of the related anion in [TcF_6_]^2−^ salts^*a*^

	*M*—F, *M* = Re	F—*M*—F, *M* = Re	*M*—F, *M* = Tc	F—*M*—F, *M* = Tc
K_2_[*M*F_6_]	1.948 (3)	86.08 (12), 93.92 (12), 180	1.928 (6)	86.93 (5), 93.07 (5), 180
Rb_2_[*M*F_6_]	1.945 (7)	86.5 (3), 93.5 (3), 180	1.933 (3)	87.2 (2), 92.8 (2), 180
Cs_2_[*M*F_6_]	1.9594 (18)	86.86 (7), 93.14 (7), 180	1.935 (5)	87.8 (2), 92.2 (2), 180

Note: (*a*) Balasekaran *et al.* (2013 ▸).

**Table 2:** Experimental details

	K_2_[ReF_6_]	Rb_2_[ReF_6_]	Cs_2_[ReF_6_]
Crystal data			
*M*_r_	378.40	471.14	566.02
Crystal system, space group	Trigonal, *P* 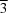 *m*1	Trigonal, *P* 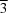 *m*1	Trigonal, *P* 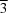 *m*1
Temperature (K)	100	100	100
*a*, *c* (Å)	5.834 (2), 4.546 (2)	5.9926 (13), 4.7177 (10)	6.268 (1), 4.931 (1)
*V* (Å^3^)	134.00 (11)	146.72 (7)	167.77 (6)
*Z*	1	1	1
Radiation type	Mo *K*α	Mo *K*α	Mo *K*α
μ (mm^−1^)	24.26	37.22	28.83
Crystal size (mm)	0.10 × 0.07 × 0.04	0.08 × 0.07 × 0.04	0.25 × 0.12 × 0.11

Data collection			
Diffractometer	Bruker D8 QUEST	Bruker D8 QUEST	Bruker D8 QUEST
Absorption correction	Multi-scan (*SADABS*; Bruker, 2015 ▸)	Numerical (*SADABS*; Bruker, 2015 ▸)	Multi-scan (*SADABS*; Bruker, 2015 ▸)
*T*_min_, *T*_max_	0.14, 0.44	0.11, 0.30	0.05, 0.15
No. of measured, independent and observed [*I* > 2σ(*I*)] reflections	2148, 180, 180	1526, 115, 111	2683, 218, 218
*R*_int_	0.054	0.073	0.040
(sin θ/λ)_max_ (Å^−1^)	0.714	0.593	0.713

Refinement			
*R*[*F* ^2^ > 2σ(*F* ^2^)], *wR*(*F* ^2^), *S*	0.016, 0.041, 1.13	0.027, 0.074, 1.30	0.013, 0.036, 1.25
No. of reflections	180	115	218
No. of parameters	12	12	13
Δρ_max_, Δρ_min_ (e Å^−3^)	1.80, −1.37	1.91, −1.36	0.68, −2.92

Computer programs: *APEX3* and *SAINT* (Bruker, 2015 ▸), *SHELXS* (Sheldrick, 2008 ▸), *SHELXL2014* (Sheldrick, 2015 ▸), *DIAMOND* (Brandenburg, 2007 ▸) and *publCIF* (Westrip, 2010 ▸).

## supplementary crystallographic information

### Dipotassium hexafluoridorhenate(IV) (SMB_K2ReF6_1) Crystal data

**Table:**

K_2_[ReF_6_]	*D*_x_ = 4.689 Mg m^−^^3^
*M**_r_* = 378.40	Mo *K*α radiation, λ = 0.71073 Å
Trigonal, *P*3*m*1	Cell parameters from 2236 reflections
*a* = 5.834 (2) Å	θ = 4.0–30.5°
*c* = 4.546 (2) Å	µ = 24.26 mm^−^^1^
*V* = 134.00 (11) Å^3^	*T* = 100 K
*Z* = 1	Hexagonal, translucent colourless
*F*(000) = 167	0.10 × 0.07 × 0.04 mm

### Dipotassium hexafluoridorhenate(IV) (SMB_K2ReF6_1) Data collection

**Table:**

Bruker D8 QUEST diffractometer	180 independent reflections
Radiation source: sealed tube, Siemens KFFMo2K-90	180 reflections with *I* > 2σ(*I*)
Curved graphite monochromator	*R*_int_ = 0.054
Detector resolution: 8.3333 pixels mm^-1^	θ_max_ = 30.5°, θ_min_ = 4.0°
φ and ω scans	*h* = −8→8
Absorption correction: multi-scan (*SADABS*; Bruker, 2015)	*k* = −8→8
*T*_min_ = 0.14, *T*_max_ = 0.44	*l* = −6→6
2148 measured reflections	

### Dipotassium hexafluoridorhenate(IV) (SMB_K2ReF6_1) Refinement

**Table:**

Refinement on *F*^2^	0 restraints
Least-squares matrix: full	Primary atom site location: structure-invariant direct methods
*R*[*F*^2^ > 2σ(*F*^2^)] = 0.016	Secondary atom site location: difference Fourier map
*wR*(*F*^2^) = 0.041	*w* = 1/[σ^2^(*F*_o_^2^) + (0.0194*P*)^2^ + 0.517*P*] where *P* = (*F*_o_^2^ + 2*F*_c_^2^)/3
*S* = 1.13	(Δ/σ)_max_ < 0.001
180 reflections	Δρ_max_ = 1.80 e Å^−^^3^
12 parameters	Δρ_min_ = −1.37 e Å^−^^3^

### Dipotassium hexafluoridorhenate(IV) (SMB_K2ReF6_1) Special details

**Table:**

Geometry. All esds (except the esd in the dihedral angle between two l.s. planes) are estimated using the full covariance matrix. The cell esds are taken into account individually in the estimation of esds in distances, angles and torsion angles; correlations between esds in cell parameters are only used when they are defined by crystal symmetry. An approximate (isotropic) treatment of cell esds is used for estimating esds involving l.s. planes.

### Dipotassium hexafluoridorhenate(IV) (SMB_K2ReF6_1) Fractional atomic coordinates and isotropic or equivalent isotropic displacement parameters (Å^2^)

**Table:**

	*x*	*y*	*z*	*U*_iso_*/*U*_eq_	
Re1	0	0	0	0.00863 (14)	
F1	0.3254 (6)	0.1627 (3)	0.2299 (6)	0.0137 (5)	
K1	0.3333	0.6667	0.2955 (4)	0.0111 (3)	

### Dipotassium hexafluoridorhenate(IV) (SMB_K2ReF6_1) Atomic displacement parameters (Å^2^)

**Table:**

	*U*^11^	*U*^22^	*U*^33^	*U*^12^	*U*^13^	*U*^23^
Re1	0.00883 (16)	0.00883 (16)	0.0082 (2)	0.00441 (8)	0	0
F1	0.0119 (13)	0.0162 (10)	0.0117 (12)	0.0060 (6)	−0.0012 (10)	−0.0006 (5)
K1	0.0118 (5)	0.0118 (5)	0.0096 (6)	0.0059 (2)	0	0

### Dipotassium hexafluoridorhenate(IV) (SMB_K2ReF6_1) Geometric parameters (Å, º)

**Table:**

Re1—F1	1.948 (3)	F1—K1^viii^	2.9325 (10)
Re1—F1^i^	1.948 (3)	F1—K1^vi^	2.946 (3)
Re1—F1^ii^	1.948 (3)	K1—F1^xi^	2.762 (3)
Re1—F1^iii^	1.948 (3)	K1—F1^x^	2.762 (3)
Re1—F1^iv^	1.948 (3)	K1—F1^xii^	2.762 (3)
Re1—F1^v^	1.948 (3)	K1—F1^xiii^	2.9325 (11)
Re1—K1^i^	3.6263 (13)	K1—F1^xiv^	2.9325 (10)
Re1—K1^vi^	3.6263 (13)	K1—F1^iv^	2.9325 (11)
Re1—K1^vii^	3.6263 (13)	K1—F1^xv^	2.9325 (11)
Re1—K1	3.6263 (13)	K1—F1^xvi^	2.9325 (11)
Re1—K1^viii^	3.6263 (13)	K1—F1^xvii^	2.946 (3)
Re1—K1^ix^	3.6263 (13)	K1—F1^ii^	2.946 (3)
F1—K1^x^	2.762 (3)	K1—F1^vi^	2.946 (3)
F1—K1	2.9325 (11)		
			
F1—Re1—F1^i^	180.0	K1—F1—K1^viii^	168.22 (13)
F1—Re1—F1^ii^	86.08 (12)	Re1—F1—K1^vi^	93.38 (11)
F1^i^—Re1—F1^ii^	93.92 (12)	K1^x^—F1—K1^vi^	105.55 (10)
F1—Re1—F1^iii^	93.92 (12)	K1—F1—K1^vi^	94.27 (6)
F1^i^—Re1—F1^iii^	86.08 (12)	K1^viii^—F1—K1^vi^	94.27 (6)
F1^ii^—Re1—F1^iii^	180.00 (19)	F1^xi^—K1—F1^x^	65.46 (11)
F1—Re1—F1^iv^	93.92 (12)	F1^xi^—K1—F1^xii^	65.46 (11)
F1^i^—Re1—F1^iv^	86.08 (12)	F1^x^—K1—F1^xii^	65.46 (11)
F1^ii^—Re1—F1^iv^	86.08 (12)	F1^xi^—K1—F1^xiii^	62.44 (10)
F1^iii^—Re1—F1^iv^	93.92 (12)	F1^x^—K1—F1^xiii^	127.81 (6)
F1—Re1—F1^v^	86.08 (12)	F1^xii^—K1—F1^xiii^	95.05 (6)
F1^i^—Re1—F1^v^	93.92 (12)	F1^xi^—K1—F1^xiv^	62.44 (10)
F1^ii^—Re1—F1^v^	93.92 (12)	F1^x^—K1—F1^xiv^	95.05 (6)
F1^iii^—Re1—F1^v^	86.08 (12)	F1^xii^—K1—F1^xiv^	127.81 (6)
F1^iv^—Re1—F1^v^	180.00 (7)	F1^xiii^—K1—F1^xiv^	58.10 (11)
F1—Re1—K1^i^	126.206 (14)	F1^xi^—K1—F1^iv^	95.05 (6)
F1^i^—Re1—K1^i^	53.794 (14)	F1^x^—K1—F1^iv^	127.81 (6)
F1^ii^—Re1—K1^i^	125.81 (9)	F1^xii^—K1—F1^iv^	62.44 (10)
F1^iii^—Re1—K1^i^	54.19 (9)	F1^xiii^—K1—F1^iv^	61.22 (12)
F1^iv^—Re1—K1^i^	126.205 (14)	F1^xiv^—K1—F1^iv^	118.98 (2)
F1^v^—Re1—K1^i^	53.795 (14)	F1^xi^—K1—F1^xv^	95.05 (6)
F1—Re1—K1^vi^	54.19 (9)	F1^x^—K1—F1^xv^	62.44 (10)
F1^i^—Re1—K1^vi^	125.81 (9)	F1^xii^—K1—F1^xv^	127.81 (6)
F1^ii^—Re1—K1^vi^	53.794 (14)	F1^xiii^—K1—F1^xv^	118.98 (2)
F1^iii^—Re1—K1^vi^	126.206 (14)	F1^xiv^—K1—F1^xv^	61.22 (12)
F1^iv^—Re1—K1^vi^	126.205 (14)	F1^iv^—K1—F1^xv^	168.22 (13)
F1^v^—Re1—K1^vi^	53.795 (14)	F1^xi^—K1—F1^xvi^	127.81 (6)
K1^i^—Re1—K1^vi^	107.11 (3)	F1^x^—K1—F1^xvi^	62.44 (10)
F1—Re1—K1^vii^	125.81 (9)	F1^xii^—K1—F1^xvi^	95.05 (6)
F1^i^—Re1—K1^vii^	54.19 (9)	F1^xiii^—K1—F1^xvi^	168.22 (13)
F1^ii^—Re1—K1^vii^	126.206 (14)	F1^xiv^—K1—F1^xvi^	118.98 (2)
F1^iii^—Re1—K1^vii^	53.794 (14)	F1^iv^—K1—F1^xvi^	118.98 (2)
F1^iv^—Re1—K1^vii^	53.795 (14)	F1^xv^—K1—F1^xvi^	58.10 (11)
F1^v^—Re1—K1^vii^	126.205 (14)	F1^xi^—K1—F1	127.81 (6)
K1^i^—Re1—K1^vii^	72.89 (3)	F1^x^—K1—F1	95.05 (6)
K1^vi^—Re1—K1^vii^	180.0	F1^xii^—K1—F1	62.44 (10)
F1—Re1—K1	53.794 (14)	F1^xiii^—K1—F1	118.98 (2)
F1^i^—Re1—K1	126.206 (14)	F1^xiv^—K1—F1	168.22 (13)
F1^ii^—Re1—K1	54.19 (9)	F1^iv^—K1—F1	58.09 (11)
F1^iii^—Re1—K1	125.81 (9)	F1^xv^—K1—F1	118.98 (2)
F1^iv^—Re1—K1	53.794 (14)	F1^xvi^—K1—F1	61.22 (11)
F1^v^—Re1—K1	126.206 (14)	F1^xi^—K1—F1^xvii^	105.55 (10)
K1^i^—Re1—K1	180.0	F1^x^—K1—F1^xvii^	144.70 (4)
K1^vi^—Re1—K1	72.89 (3)	F1^xii^—K1—F1^xvii^	144.70 (4)
K1^vii^—Re1—K1	107.11 (3)	F1^xiii^—K1—F1^xvii^	53.80 (10)
F1—Re1—K1^viii^	53.795 (14)	F1^xiv^—K1—F1^xvii^	53.80 (10)
F1^i^—Re1—K1^viii^	126.205 (14)	F1^iv^—K1—F1^xvii^	85.73 (6)
F1^ii^—Re1—K1^viii^	126.206 (14)	F1^xv^—K1—F1^xvii^	85.73 (6)
F1^iii^—Re1—K1^viii^	53.794 (14)	F1^xvi^—K1—F1^xvii^	114.69 (6)
F1^iv^—Re1—K1^viii^	125.81 (9)	F1—K1—F1^xvii^	114.69 (6)
F1^v^—Re1—K1^viii^	54.19 (9)	F1^xi^—K1—F1^ii^	144.70 (4)
K1^i^—Re1—K1^viii^	72.90 (3)	F1^x^—K1—F1^ii^	144.70 (4)
K1^vi^—Re1—K1^viii^	72.90 (3)	F1^xii^—K1—F1^ii^	105.55 (10)
K1^vii^—Re1—K1^viii^	107.10 (3)	F1^xiii^—K1—F1^ii^	85.73 (6)
K1—Re1—K1^viii^	107.10 (3)	F1^xiv^—K1—F1^ii^	114.69 (6)
F1—Re1—K1^ix^	126.205 (14)	F1^iv^—K1—F1^ii^	53.80 (10)
F1^i^—Re1—K1^ix^	53.795 (14)	F1^xv^—K1—F1^ii^	114.69 (6)
F1^ii^—Re1—K1^ix^	53.794 (14)	F1^xvi^—K1—F1^ii^	85.73 (6)
F1^iii^—Re1—K1^ix^	126.206 (14)	F1—K1—F1^ii^	53.80 (10)
F1^iv^—Re1—K1^ix^	54.19 (9)	F1^xvii^—K1—F1^ii^	60.91 (10)
F1^v^—Re1—K1^ix^	125.81 (9)	F1^xi^—K1—F1^vi^	144.70 (4)
K1^i^—Re1—K1^ix^	107.10 (3)	F1^x^—K1—F1^vi^	105.55 (10)
K1^vi^—Re1—K1^ix^	107.10 (3)	F1^xii^—K1—F1^vi^	144.70 (4)
K1^vii^—Re1—K1^ix^	72.90 (3)	F1^xiii^—K1—F1^vi^	114.69 (6)
K1—Re1—K1^ix^	72.90 (3)	F1^xiv^—K1—F1^vi^	85.73 (6)
K1^viii^—Re1—K1^ix^	180.0	F1^iv^—K1—F1^vi^	114.69 (6)
Re1—F1—K1^x^	161.08 (14)	F1^xv^—K1—F1^vi^	53.80 (10)
Re1—F1—K1	93.79 (6)	F1^xvi^—K1—F1^vi^	53.80 (10)
K1^x^—F1—K1	84.95 (6)	F1—K1—F1^vi^	85.73 (6)
Re1—F1—K1^viii^	93.79 (6)	F1^xvii^—K1—F1^vi^	60.91 (10)
K1^x^—F1—K1^viii^	84.95 (6)	F1^ii^—K1—F1^vi^	60.91 (10)

Symmetry codes: (i) −*x*, −*y*, −*z*; (ii) *x*−*y*, *x*, −*z*; (iii) −*x*+*y*, −*x*, *z*; (iv) −*y*, *x*−*y*, *z*; (v) *y*, −*x*+*y*, −*z*; (vi) −*x*+1, −*y*+1, −*z*; (vii) *x*−1, *y*−1, *z*; (viii) *x*, *y*−1, *z*; (ix) −*x*, −*y*+1, −*z*; (x) −*x*+1, −*y*+1, −*z*+1; (xi) *y*, −*x*+*y*+1, −*z*+1; (xii) *x*−*y*, *x*, −*z*+1; (xiii) −*x*+*y*, −*x*+1, *z*; (xiv) *x*, *y*+1, *z*; (xv) −*y*+1, *x*−*y*+1, *z*; (xvi) −*x*+*y*+1, −*x*+1, *z*; (xvii) *y*, −*x*+*y*+1, −*z*.

### Dirubidium hexafluoridorhenate(IV) (SMB_Rb2ReF6_j) Crystal data

**Table:**

Rb_2_[ReF_6_]	*D*_x_ = 5.332 Mg m^−^^3^
*M**_r_* = 471.14	Mo *K*α radiation, λ = 0.71073 Å
Trigonal, *P*3*m*1	Cell parameters from 1612 reflections
*a* = 5.9926 (13) Å	θ = 3.9–28.3°
*c* = 4.7177 (10) Å	µ = 37.22 mm^−^^1^
*V* = 146.72 (7) Å^3^	*T* = 100 K
*Z* = 1	Hexagonal plate, translucent colourless
*F*(000) = 203	0.08 × 0.07 × 0.04 mm

### Dirubidium hexafluoridorhenate(IV) (SMB_Rb2ReF6_j) Data collection

**Table:**

Bruker D8 QUEST diffractometer	115 independent reflections
Radiation source: sealed tube, Siemens KFFMo2K-90	111 reflections with *I* > 2σ(*I*)
Curved graphite monochromator	*R*_int_ = 0.073
Detector resolution: 8.3333 pixels mm^-1^	θ_max_ = 24.9°, θ_min_ = 3.9°
φ and ω scans	*h* = −7→7
Absorption correction: numerical (*SADABS*; Bruker, 2015)	*k* = −7→7
*T*_min_ = 0.11, *T*_max_ = 0.30	*l* = −5→5
1526 measured reflections	

### Dirubidium hexafluoridorhenate(IV) (SMB_Rb2ReF6_j) Refinement

**Table:**

Refinement on *F*^2^	0 restraints
Least-squares matrix: full	Primary atom site location: heavy-atom method
*R*[*F*^2^ > 2σ(*F*^2^)] = 0.027	Secondary atom site location: difference Fourier map
*wR*(*F*^2^) = 0.074	*w* = 1/[σ^2^(*F*_o_^2^) + (0.0175*P*)^2^ + 3.5548*P*] where *P* = (*F*_o_^2^ + 2*F*_c_^2^)/3
*S* = 1.30	(Δ/σ)_max_ < 0.001
115 reflections	Δρ_max_ = 1.91 e Å^−^^3^
12 parameters	Δρ_min_ = −1.36 e Å^−^^3^

### Dirubidium hexafluoridorhenate(IV) (SMB_Rb2ReF6_j) Special details

**Table:**

Geometry. All esds (except the esd in the dihedral angle between two l.s. planes) are estimated using the full covariance matrix. The cell esds are taken into account individually in the estimation of esds in distances, angles and torsion angles; correlations between esds in cell parameters are only used when they are defined by crystal symmetry. An approximate (isotropic) treatment of cell esds is used for estimating esds involving l.s. planes.

### Dirubidium hexafluoridorhenate(IV) (SMB_Rb2ReF6_j) Fractional atomic coordinates and isotropic or equivalent isotropic displacement parameters (Å^2^)

**Table:**

	*x*	*y*	*z*	*U*_iso_*/*U*_eq_	
Re1	0	0	0	0.0157 (5)	
F1	0.3151 (14)	0.1576 (7)	0.2231 (15)	0.0151 (17)	
Rb1	0.3333	0.6667	0.2971 (4)	0.0138 (6)	

### Dirubidium hexafluoridorhenate(IV) (SMB_Rb2ReF6_j) Atomic displacement parameters (Å^2^)

**Table:**

	*U*^11^	*U*^22^	*U*^33^	*U*^12^	*U*^13^	*U*^23^
Re1	0.0133 (6)	0.0133 (6)	0.0205 (8)	0.0066 (3)	0	0
F1	0.015 (4)	0.017 (3)	0.012 (3)	0.0077 (19)	0.001 (3)	0.0007 (14)
Rb1	0.0131 (8)	0.0131 (8)	0.0152 (12)	0.0065 (4)	0	0

### Dirubidium hexafluoridorhenate(IV) (SMB_Rb2ReF6_j) Geometric parameters (Å, º)

**Table:**

Re1—F1^i^	1.945 (7)	F1—Rb1^viii^	3.0181 (11)
Re1—F1^ii^	1.945 (7)	F1—Rb1^vi^	3.058 (7)
Re1—F1^iii^	1.945 (7)	Rb1—F1^xi^	2.907 (7)
Re1—F1^iv^	1.945 (7)	Rb1—F1^x^	2.907 (7)
Re1—F1^v^	1.945 (7)	Rb1—F1^xii^	2.907 (7)
Re1—F1	1.945 (7)	Rb1—F1^xiii^	3.0181 (11)
Re1—Rb1^i^	3.7330 (10)	Rb1—F1^xiv^	3.0181 (11)
Re1—Rb1^vi^	3.7330 (11)	Rb1—F1^v^	3.0181 (11)
Re1—Rb1^vii^	3.7330 (11)	Rb1—F1^xv^	3.0181 (11)
Re1—Rb1	3.7330 (10)	Rb1—F1^xvi^	3.0181 (11)
Re1—Rb1^viii^	3.7330 (11)	Rb1—F1^xvii^	3.058 (7)
Re1—Rb1^ix^	3.7330 (11)	Rb1—F1^ii^	3.058 (7)
F1—Rb1^x^	2.907 (7)	Rb1—F1^vi^	3.058 (7)
F1—Rb1	3.0181 (11)		
			
F1^i^—Re1—F1^ii^	93.5 (3)	Rb1—F1—Rb1^viii^	166.2 (3)
F1^i^—Re1—F1^iii^	86.5 (3)	Re1—F1—Rb1^vi^	93.9 (2)
F1^ii^—Re1—F1^iii^	180.0 (3)	Rb1^x^—F1—Rb1^vi^	104.5 (2)
F1^i^—Re1—F1^iv^	93.5 (3)	Rb1—F1—Rb1^vi^	94.26 (14)
F1^ii^—Re1—F1^iv^	93.5 (3)	Rb1^viii^—F1—Rb1^vi^	94.26 (14)
F1^iii^—Re1—F1^iv^	86.5 (3)	F1^xi^—Rb1—F1^x^	65.8 (2)
F1^i^—Re1—F1^v^	86.5 (3)	F1^xi^—Rb1—F1^xii^	65.8 (2)
F1^ii^—Re1—F1^v^	86.5 (3)	F1^x^—Rb1—F1^xii^	65.8 (2)
F1^iii^—Re1—F1^v^	93.5 (3)	F1^xi^—Rb1—F1^xiii^	62.7 (2)
F1^iv^—Re1—F1^v^	180.0	F1^x^—Rb1—F1^xiii^	128.31 (8)
F1^i^—Re1—F1	180.0	F1^xii^—Rb1—F1^xiii^	96.30 (14)
F1^ii^—Re1—F1	86.5 (3)	F1^xi^—Rb1—F1^xiv^	62.7 (2)
F1^iii^—Re1—F1	93.5 (3)	F1^x^—Rb1—F1^xiv^	96.30 (14)
F1^iv^—Re1—F1	86.5 (3)	F1^xii^—Rb1—F1^xiv^	128.31 (8)
F1^v^—Re1—F1	93.5 (3)	F1^xiii^—Rb1—F1^xiv^	56.0 (3)
F1^i^—Re1—Rb1^i^	53.64 (2)	F1^xi^—Rb1—F1^v^	96.30 (14)
F1^ii^—Re1—Rb1^i^	125.2 (2)	F1^x^—Rb1—F1^v^	128.31 (8)
F1^iii^—Re1—Rb1^i^	54.8 (2)	F1^xii^—Rb1—F1^v^	62.7 (2)
F1^iv^—Re1—Rb1^i^	53.64 (2)	F1^xiii^—Rb1—F1^v^	63.1 (3)
F1^v^—Re1—Rb1^i^	126.36 (2)	F1^xiv^—Rb1—F1^v^	118.68 (6)
F1—Re1—Rb1^i^	126.36 (2)	F1^xi^—Rb1—F1^xv^	96.30 (14)
F1^i^—Re1—Rb1^vi^	125.2 (2)	F1^x^—Rb1—F1^xv^	62.7 (2)
F1^ii^—Re1—Rb1^vi^	53.64 (2)	F1^xii^—Rb1—F1^xv^	128.31 (8)
F1^iii^—Re1—Rb1^vi^	126.36 (2)	F1^xiii^—Rb1—F1^xv^	118.68 (5)
F1^iv^—Re1—Rb1^vi^	53.64 (2)	F1^xiv^—Rb1—F1^xv^	63.1 (3)
F1^v^—Re1—Rb1^vi^	126.36 (2)	F1^v^—Rb1—F1^xv^	166.2 (3)
F1—Re1—Rb1^vi^	54.8 (2)	F1^xi^—Rb1—F1^xvi^	128.31 (8)
Rb1^i^—Re1—Rb1^vi^	106.77 (3)	F1^x^—Rb1—F1^xvi^	62.7 (2)
F1^i^—Re1—Rb1^vii^	54.8 (2)	F1^xii^—Rb1—F1^xvi^	96.30 (14)
F1^ii^—Re1—Rb1^vii^	126.36 (2)	F1^xiii^—Rb1—F1^xvi^	166.2 (3)
F1^iii^—Re1—Rb1^vii^	53.64 (2)	F1^xiv^—Rb1—F1^xvi^	118.68 (6)
F1^iv^—Re1—Rb1^vii^	126.36 (2)	F1^v^—Rb1—F1^xvi^	118.68 (5)
F1^v^—Re1—Rb1^vii^	53.64 (2)	F1^xv^—Rb1—F1^xvi^	56.0 (3)
F1—Re1—Rb1^vii^	125.2 (2)	F1^xi^—Rb1—F1	128.31 (8)
Rb1^i^—Re1—Rb1^vii^	73.23 (3)	F1^x^—Rb1—F1	96.30 (14)
Rb1^vi^—Re1—Rb1^vii^	180.0	F1^xii^—Rb1—F1	62.7 (2)
F1^i^—Re1—Rb1	126.36 (2)	F1^xiii^—Rb1—F1	118.68 (6)
F1^ii^—Re1—Rb1	54.8 (2)	F1^xiv^—Rb1—F1	166.2 (3)
F1^iii^—Re1—Rb1	125.2 (2)	F1^v^—Rb1—F1	56.0 (3)
F1^iv^—Re1—Rb1	126.36 (2)	F1^xv^—Rb1—F1	118.68 (5)
F1^v^—Re1—Rb1	53.64 (2)	F1^xvi^—Rb1—F1	63.1 (3)
F1—Re1—Rb1	53.64 (2)	F1^xi^—Rb1—F1^xvii^	104.5 (2)
Rb1^i^—Re1—Rb1	180.0	F1^x^—Rb1—F1^xvii^	144.30 (9)
Rb1^vi^—Re1—Rb1	73.23 (3)	F1^xii^—Rb1—F1^xvii^	144.30 (9)
Rb1^vii^—Re1—Rb1	106.77 (3)	F1^xiii^—Rb1—F1^xvii^	52.0 (2)
F1^i^—Re1—Rb1^viii^	126.36 (2)	F1^xiv^—Rb1—F1^xvii^	52.0 (2)
F1^ii^—Re1—Rb1^viii^	126.36 (2)	F1^v^—Rb1—F1^xvii^	85.74 (14)
F1^iii^—Re1—Rb1^viii^	53.64 (2)	F1^xv^—Rb1—F1^xvii^	85.74 (14)
F1^iv^—Re1—Rb1^viii^	54.8 (2)	F1^xvi^—Rb1—F1^xvii^	114.25 (9)
F1^v^—Re1—Rb1^viii^	125.2 (2)	F1—Rb1—F1^xvii^	114.25 (9)
F1—Re1—Rb1^viii^	53.64 (2)	F1^xi^—Rb1—F1^ii^	144.30 (9)
Rb1^i^—Re1—Rb1^viii^	73.23 (3)	F1^x^—Rb1—F1^ii^	144.30 (9)
Rb1^vi^—Re1—Rb1^viii^	73.23 (3)	F1^xii^—Rb1—F1^ii^	104.5 (2)
Rb1^vii^—Re1—Rb1^viii^	106.77 (3)	F1^xiii^—Rb1—F1^ii^	85.74 (14)
Rb1—Re1—Rb1^viii^	106.77 (3)	F1^xiv^—Rb1—F1^ii^	114.25 (9)
F1^i^—Re1—Rb1^ix^	53.64 (2)	F1^v^—Rb1—F1^ii^	52.0 (2)
F1^ii^—Re1—Rb1^ix^	53.64 (2)	F1^xv^—Rb1—F1^ii^	114.25 (9)
F1^iii^—Re1—Rb1^ix^	126.36 (2)	F1^xvi^—Rb1—F1^ii^	85.74 (14)
F1^iv^—Re1—Rb1^ix^	125.2 (2)	F1—Rb1—F1^ii^	52.0 (2)
F1^v^—Re1—Rb1^ix^	54.8 (2)	F1^xvii^—Rb1—F1^ii^	62.2 (2)
F1—Re1—Rb1^ix^	126.36 (2)	F1^xi^—Rb1—F1^vi^	144.30 (9)
Rb1^i^—Re1—Rb1^ix^	106.77 (3)	F1^x^—Rb1—F1^vi^	104.5 (2)
Rb1^vi^—Re1—Rb1^ix^	106.77 (3)	F1^xii^—Rb1—F1^vi^	144.30 (9)
Rb1^vii^—Re1—Rb1^ix^	73.23 (3)	F1^xiii^—Rb1—F1^vi^	114.25 (9)
Rb1—Re1—Rb1^ix^	73.23 (3)	F1^xiv^—Rb1—F1^vi^	85.74 (14)
Rb1^viii^—Re1—Rb1^ix^	180.0	F1^v^—Rb1—F1^vi^	114.25 (9)
Re1—F1—Rb1^x^	161.6 (3)	F1^xv^—Rb1—F1^vi^	52.0 (2)
Re1—F1—Rb1	95.10 (14)	F1^xvi^—Rb1—F1^vi^	52.0 (2)
Rb1^x^—F1—Rb1	83.70 (14)	F1—Rb1—F1^vi^	85.74 (14)
Re1—F1—Rb1^viii^	95.10 (14)	F1^xvii^—Rb1—F1^vi^	62.2 (2)
Rb1^x^—F1—Rb1^viii^	83.70 (14)	F1^ii^—Rb1—F1^vi^	62.2 (2)

Symmetry codes: (i) −*x*, −*y*, −*z*; (ii) *x*−*y*, *x*, −*z*; (iii) −*x*+*y*, −*x*, *z*; (iv) *y*, −*x*+*y*, −*z*; (v) −*y*, *x*−*y*, *z*; (vi) −*x*+1, −*y*+1, −*z*; (vii) *x*−1, *y*−1, *z*; (viii) *x*, *y*−1, *z*; (ix) −*x*, −*y*+1, −*z*; (x) −*x*+1, −*y*+1, −*z*+1; (xi) *y*, −*x*+*y*+1, −*z*+1; (xii) *x*−*y*, *x*, −*z*+1; (xiii) −*x*+*y*, −*x*+1, *z*; (xiv) *x*, *y*+1, *z*; (xv) −*y*+1, *x*−*y*+1, *z*; (xvi) −*x*+*y*+1, −*x*+1, *z*; (xvii) *y*, −*x*+*y*+1, −*z*.

### Dicaesium hexafluoridorhenate(IV) (SMB_Cs2ReF6_1a) Crystal data

**Table:**

Cs_2_[ReF_6_]	*D*_x_ = 5.602 Mg m^−^^3^
*M**_r_* = 566.02	Mo *K*α radiation, λ = 0.71073 Å
Trigonal, *P*3*m*1	Cell parameters from 135 reflections
*a* = 6.268 (1) Å	θ = 3.8–32.6°
*c* = 4.931 (1) Å	µ = 28.83 mm^−^^1^
*V* = 167.77 (6) Å^3^	*T* = 100 K
*Z* = 1	Hexagonal plate, clear colourless
*F*(000) = 239	0.25 × 0.12 × 0.11 mm

### Dicaesium hexafluoridorhenate(IV) (SMB_Cs2ReF6_1a) Data collection

**Table:**

Bruker D8 QUEST diffractometer	218 independent reflections
Radiation source: sealed tube, Siemens KFFMo2K-90	218 reflections with *I* > 2σ(*I*)
Curved graphite monochromator	*R*_int_ = 0.040
Detector resolution: 8.3333 pixels mm^-1^	θ_max_ = 30.4°, θ_min_ = 3.8°
φ and ω scans	*h* = −8→8
Absorption correction: multi-scan (*SADABS*; Bruker, 2015)	*k* = −8→8
*T*_min_ = 0.05, *T*_max_ = 0.15	*l* = −7→7
2683 measured reflections	

### Dicaesium hexafluoridorhenate(IV) (SMB_Cs2ReF6_1a) Refinement

**Table:**

Refinement on *F*^2^	Primary atom site location: structure-invariant direct methods
Least-squares matrix: full	Secondary atom site location: difference Fourier map
*R*[*F*^2^ > 2σ(*F*^2^)] = 0.013	*w* = 1/[σ^2^(*F*_o_^2^) + (0.0181*P*)^2^ + 0.1419*P*] where *P* = (*F*_o_^2^ + 2*F*_c_^2^)/3
*wR*(*F*^2^) = 0.036	(Δ/σ)_max_ < 0.001
*S* = 1.25	Δρ_max_ = 0.68 e Å^−^^3^
218 reflections	Δρ_min_ = −2.92 e Å^−^^3^
13 parameters	Extinction correction: SHELXL2014 (Sheldrick, 2015)
0 restraints	Extinction coefficient: 0.029 (2)

### Dicaesium hexafluoridorhenate(IV) (SMB_Cs2ReF6_1a) Special details

**Table:**

Geometry. All esds (except the esd in the dihedral angle between two l.s. planes) are estimated using the full covariance matrix. The cell esds are taken into account individually in the estimation of esds in distances, angles and torsion angles; correlations between esds in cell parameters are only used when they are defined by crystal symmetry. An approximate (isotropic) treatment of cell esds is used for estimating esds involving l.s. planes.

### Dicaesium hexafluoridorhenate(IV) (SMB_Cs2ReF6_1a) Fractional atomic coordinates and isotropic or equivalent isotropic displacement parameters (Å^2^)

**Table:**

	*x*	*y*	*z*	*U*_iso_*/*U*_eq_	
Re1	0	0	0	0.00427 (15)	
F1	0.3027 (3)	0.15135 (17)	0.2165 (4)	0.0092 (4)	
Cs1	0.3333	0.6667	0.30027 (9)	0.00615 (14)	

### Dicaesium hexafluoridorhenate(IV) (SMB_Cs2ReF6_1a) Atomic displacement parameters (Å^2^)

**Table:**

	*U*^11^	*U*^22^	*U*^33^	*U*^12^	*U*^13^	*U*^23^
Re1	0.00483 (16)	0.00483 (16)	0.0032 (2)	0.00241 (8)	0	0
F1	0.0088 (8)	0.0107 (6)	0.0075 (9)	0.0044 (4)	−0.0023 (6)	−0.0012 (3)
Cs1	0.00661 (16)	0.00661 (16)	0.0052 (2)	0.00331 (8)	0	0

### Dicaesium hexafluoridorhenate(IV) (SMB_Cs2ReF6_1a) Geometric parameters (Å, º)

**Table:**

Re1—F1^i^	1.9594 (18)	F1—Cs1^viii^	3.1655 (6)
Re1—F1^ii^	1.9594 (18)	F1—Cs1^vi^	3.224 (2)
Re1—F1^iii^	1.9594 (18)	Cs1—F1^xi^	3.0955 (19)
Re1—F1^iv^	1.9594 (18)	Cs1—F1^x^	3.0955 (19)
Re1—F1^v^	1.9594 (18)	Cs1—F1^xii^	3.0955 (19)
Re1—F1	1.9594 (18)	Cs1—F1^xiii^	3.1655 (6)
Re1—Cs1^i^	3.9100 (6)	Cs1—F1^xiv^	3.1655 (6)
Re1—Cs1^vi^	3.9100 (6)	Cs1—F1^iii^	3.1655 (6)
Re1—Cs1^vii^	3.9100 (6)	Cs1—F1^xv^	3.1655 (6)
Re1—Cs1	3.9100 (6)	Cs1—F1^xvi^	3.1655 (6)
Re1—Cs1^viii^	3.9100 (6)	Cs1—F1^xvii^	3.224 (2)
Re1—Cs1^ix^	3.9100 (6)	Cs1—F1^iv^	3.224 (2)
F1—Cs1^x^	3.0955 (19)	Cs1—F1^vi^	3.224 (2)
F1—Cs1	3.1655 (6)		
			
F1^i^—Re1—F1^ii^	93.14 (7)	Cs1—F1—Cs1^viii^	163.82 (6)
F1^i^—Re1—F1^iii^	86.86 (7)	Re1—F1—Cs1^vi^	94.78 (7)
F1^ii^—Re1—F1^iii^	180.00 (4)	Cs1^x^—F1—Cs1^vi^	102.55 (5)
F1^i^—Re1—F1^iv^	93.14 (7)	Cs1—F1—Cs1^vi^	94.07 (3)
F1^ii^—Re1—F1^iv^	93.14 (7)	Cs1^viii^—F1—Cs1^vi^	94.07 (3)
F1^iii^—Re1—F1^iv^	86.86 (7)	F1^xi^—Cs1—F1^x^	67.11 (6)
F1^i^—Re1—F1^v^	86.86 (7)	F1^xi^—Cs1—F1^xii^	67.11 (6)
F1^ii^—Re1—F1^v^	86.86 (7)	F1^x^—Cs1—F1^xii^	67.11 (6)
F1^iii^—Re1—F1^v^	93.14 (7)	F1^xi^—Cs1—F1^xiii^	62.38 (6)
F1^iv^—Re1—F1^v^	180.00 (8)	F1^x^—Cs1—F1^xiii^	129.122 (17)
F1^i^—Re1—F1	180.0	F1^xii^—Cs1—F1^xiii^	97.70 (3)
F1^ii^—Re1—F1	86.86 (7)	F1^xi^—Cs1—F1^xiv^	62.38 (6)
F1^iii^—Re1—F1	93.14 (7)	F1^x^—Cs1—F1^xiv^	97.70 (3)
F1^iv^—Re1—F1	86.86 (7)	F1^xii^—Cs1—F1^xiv^	129.122 (17)
F1^v^—Re1—F1	93.14 (7)	F1^xiii^—Cs1—F1^xiv^	53.43 (7)
F1^i^—Re1—Cs1^i^	53.533 (6)	F1^xi^—Cs1—F1^iii^	97.70 (3)
F1^ii^—Re1—Cs1^i^	53.533 (6)	F1^x^—Cs1—F1^iii^	129.122 (17)
F1^iii^—Re1—Cs1^i^	126.467 (6)	F1^xii^—Cs1—F1^iii^	62.38 (6)
F1^iv^—Re1—Cs1^i^	124.74 (6)	F1^xiii^—Cs1—F1^iii^	65.44 (7)
F1^v^—Re1—Cs1^i^	55.26 (6)	F1^xiv^—Cs1—F1^iii^	118.323 (15)
F1—Re1—Cs1^i^	126.467 (6)	F1^xi^—Cs1—F1^xv^	97.70 (3)
F1^i^—Re1—Cs1^vi^	124.74 (6)	F1^x^—Cs1—F1^xv^	62.38 (6)
F1^ii^—Re1—Cs1^vi^	53.533 (6)	F1^xii^—Cs1—F1^xv^	129.122 (17)
F1^iii^—Re1—Cs1^vi^	126.467 (6)	F1^xiii^—Cs1—F1^xv^	118.323 (14)
F1^iv^—Re1—Cs1^vi^	53.532 (6)	F1^xiv^—Cs1—F1^xv^	65.44 (7)
F1^v^—Re1—Cs1^vi^	126.468 (6)	F1^iii^—Cs1—F1^xv^	163.82 (6)
F1—Re1—Cs1^vi^	55.26 (6)	F1^xi^—Cs1—F1	129.123 (17)
Cs1^i^—Re1—Cs1^vi^	106.554 (9)	F1^x^—Cs1—F1	97.70 (3)
F1^i^—Re1—Cs1^vii^	55.26 (6)	F1^xii^—Cs1—F1	62.38 (6)
F1^ii^—Re1—Cs1^vii^	126.467 (6)	F1^xiii^—Cs1—F1	118.323 (15)
F1^iii^—Re1—Cs1^vii^	53.533 (6)	F1^xiv^—Cs1—F1	163.82 (6)
F1^iv^—Re1—Cs1^vii^	126.468 (6)	F1^iii^—Cs1—F1	53.43 (7)
F1^v^—Re1—Cs1^vii^	53.532 (6)	F1^xv^—Cs1—F1	118.323 (15)
F1—Re1—Cs1^vii^	124.74 (6)	F1^xi^—Cs1—F1^xvi^	129.122 (17)
Cs1^i^—Re1—Cs1^vii^	73.446 (9)	F1^x^—Cs1—F1^xvi^	62.38 (6)
Cs1^vi^—Re1—Cs1^vii^	180.0	F1^xii^—Cs1—F1^xvi^	97.70 (3)
F1^i^—Re1—Cs1	126.467 (6)	F1^xiii^—Cs1—F1^xvi^	163.82 (6)
F1^ii^—Re1—Cs1	126.467 (6)	F1^xiv^—Cs1—F1^xvi^	118.323 (15)
F1^iii^—Re1—Cs1	53.533 (6)	F1^iii^—Cs1—F1^xvi^	118.323 (15)
F1^iv^—Re1—Cs1	55.26 (6)	F1^xv^—Cs1—F1^xvi^	53.43 (7)
F1^v^—Re1—Cs1	124.74 (6)	F1—Cs1—F1^xvi^	65.44 (7)
F1—Re1—Cs1	53.533 (6)	F1^xi^—Cs1—F1^xvii^	102.55 (5)
Cs1^i^—Re1—Cs1	180.0	F1^x^—Cs1—F1^xvii^	143.51 (2)
Cs1^vi^—Re1—Cs1	73.447 (8)	F1^xii^—Cs1—F1^xvii^	143.51 (2)
Cs1^vii^—Re1—Cs1	106.553 (8)	F1^xiii^—Cs1—F1^xvii^	49.86 (5)
F1^i^—Re1—Cs1^viii^	126.467 (6)	F1^xiv^—Cs1—F1^xvii^	49.86 (5)
F1^ii^—Re1—Cs1^viii^	55.26 (6)	F1^iii^—Cs1—F1^xvii^	85.93 (3)
F1^iii^—Re1—Cs1^viii^	124.74 (6)	F1^xv^—Cs1—F1^xvii^	85.93 (3)
F1^iv^—Re1—Cs1^viii^	126.467 (6)	F1—Cs1—F1^xvii^	113.96 (2)
F1^v^—Re1—Cs1^viii^	53.533 (6)	F1^xvi^—Cs1—F1^xvii^	113.96 (2)
F1—Re1—Cs1^viii^	53.533 (6)	F1^xi^—Cs1—F1^iv^	143.51 (2)
Cs1^i^—Re1—Cs1^viii^	73.447 (8)	F1^x^—Cs1—F1^iv^	143.51 (2)
Cs1^vi^—Re1—Cs1^viii^	73.447 (9)	F1^xii^—Cs1—F1^iv^	102.55 (5)
Cs1^vii^—Re1—Cs1^viii^	106.553 (9)	F1^xiii^—Cs1—F1^iv^	85.93 (3)
Cs1—Re1—Cs1^viii^	106.553 (9)	F1^xiv^—Cs1—F1^iv^	113.96 (2)
F1^i^—Re1—Cs1^ix^	53.533 (6)	F1^iii^—Cs1—F1^iv^	49.86 (5)
F1^ii^—Re1—Cs1^ix^	124.74 (6)	F1^xv^—Cs1—F1^iv^	113.96 (2)
F1^iii^—Re1—Cs1^ix^	55.26 (6)	F1—Cs1—F1^iv^	49.86 (5)
F1^iv^—Re1—Cs1^ix^	53.533 (6)	F1^xvi^—Cs1—F1^iv^	85.93 (3)
F1^v^—Re1—Cs1^ix^	126.467 (6)	F1^xvii^—Cs1—F1^iv^	64.10 (5)
F1—Re1—Cs1^ix^	126.467 (6)	F1^xi^—Cs1—F1^vi^	143.51 (2)
Cs1^i^—Re1—Cs1^ix^	106.553 (8)	F1^x^—Cs1—F1^vi^	102.55 (5)
Cs1^vi^—Re1—Cs1^ix^	106.553 (9)	F1^xii^—Cs1—F1^vi^	143.51 (2)
Cs1^vii^—Re1—Cs1^ix^	73.447 (9)	F1^xiii^—Cs1—F1^vi^	113.96 (2)
Cs1—Re1—Cs1^ix^	73.447 (9)	F1^xiv^—Cs1—F1^vi^	85.93 (3)
Cs1^viii^—Re1—Cs1^ix^	180.0	F1^iii^—Cs1—F1^vi^	113.96 (2)
Re1—F1—Cs1^x^	162.67 (9)	F1^xv^—Cs1—F1^vi^	49.86 (5)
Re1—F1—Cs1	96.61 (3)	F1—Cs1—F1^vi^	85.93 (3)
Cs1^x^—F1—Cs1	82.30 (3)	F1^xvi^—Cs1—F1^vi^	49.86 (5)
Re1—F1—Cs1^viii^	96.61 (3)	F1^xvii^—Cs1—F1^vi^	64.10 (5)
Cs1^x^—F1—Cs1^viii^	82.30 (3)	F1^iv^—Cs1—F1^vi^	64.10 (5)

Symmetry codes: (i) −*x*, −*y*, −*z*; (ii) *y*, −*x*+*y*, −*z*; (iii) −*y*, *x*−*y*, *z*; (iv) *x*−*y*, *x*, −*z*; (v) −*x*+*y*, −*x*, *z*; (vi) −*x*+1, −*y*+1, −*z*; (vii) *x*−1, *y*−1, *z*; (viii) *x*, *y*−1, *z*; (ix) −*x*, −*y*+1, −*z*; (x) −*x*+1, −*y*+1, −*z*+1; (xi) *y*, −*x*+*y*+1, −*z*+1; (xii) *x*−*y*, *x*, −*z*+1; (xiii) −*x*+*y*, −*x*+1, *z*; (xiv) *x*, *y*+1, *z*; (xv) −*y*+1, *x*−*y*+1, *z*; (xvi) −*x*+*y*+1, −*x*+1, *z*; (xvii) *y*, −*x*+*y*+1, −*z*.

## References

[bb1] Balasekaran, S. M., Molski, M., Spandl, J., Hagenbach, A., Alberto, R. & Abram, U. (2013). *Inorg. Chem.* **52**, 7094–7099. 10.1021/ic400775e 23713911

[bb2] Bettinelli, M., Disipio, L., Ingletto, G. & Razzetti, C. (1987). *Inorg. Chim. Acta*, **133**, 7–9.

[bb3] Brandenburg, K. (2007). *DIAMOND.* Crystal Impact GbR, Bonn, Germany.

[bb4] Brauer, G. & Allardt, H. D. (1962). *Z. Anorg. Allg. Chem.* **316**, 134–140.

[bb5] Bruker (2015). *APEX3*, *SAINT* and *SADABS*. Bruker AXS Inc., Madison, WI, USA.

[bb6] Clark, G. R. & Russell, D. R. (1978). *Acta Cryst.* B**34**, 894–895.

[bb7] Hoard, J. L. & Vincent, W. B. (1939). *J. Am. Chem. Soc.* **61**, 2849–2852.

[bb8] Peacock, R. D. (1956). *J. Chem. Soc.* pp. 1291–1293.

[bb9] Pedersen, K. S., Sigrist, M., Sorensen, M. A., Barra, A. L., Weyhermuller, T., Piligkos, S., Thuesen, C. A., Vinum, M. G., Mutka, H., Weihe, H., Clerac, R. & Bendix, J. (2014). *Angew. Chem. Int. Ed.* **53**, 1351–1354. 10.1002/anie.201309981 24459056

[bb10] Ruff, O. & Kwasnik, W. (1934). *Z. Anorg. Allg. Chem.* **219**, 65–81.

[bb11] Schwochau, K. & Herr, W. (1963). *Angew. Chem.* **75**, 95.

[bb12] Sheldrick, G. M. (2008). *Acta Cryst.* A**64**, 112–122. 10.1107/S0108767307043930 18156677

[bb13] Sheldrick, G. M. (2015). *Acta Cryst.* C**71**, 3–8.

[bb16] Watt, G. W., Thompson, R. J. & Gibbons, J. M. (1963). *Inorganic Syntheses* edited by J. Kleinberg, Vol 7, pp. 189–190. New York: McGraw-Hill.

[bb14] Weise, E. (1956). *Z. Anorg. Allg. Chem.* **283**, 377–389.

[bb15] Westrip, S. P. (2010). *J. Appl. Cryst.* **43**, 920–925.

