# Supplementary material for: Syntheses, Raman spectroscopy and crystal structures of alkali hexa­fluorido­rhenates(IV) revisited
Source: Acta Crystallogr E Crystallogr Commun. 2018 Apr 6;74(Pt 5):646–9. doi: 10.1107/S2056989018005297 (PMC5947479; doi:10.1107/S2056989018005297)
Supplement: Supplementary file 6 [file e-74-00646-sup6.pdf]

Supporting information

Hexafluororhenate(IV) – revisited

James Louis-Jean†, Samundeeswari Mariappan Balasekaran†, Dean Smith‡, Ashkan Salamat‡, Thang Chien Pham‡, Frederic Poineau†

Experimental Section
Materials

Ammonium perrhenate, ammonium bifluoride, potassium fluoride, rubidium fluoride, cesium fluoride, and hydrobromic acid (48%) were purchased from Sigma Aldrich and used without any further purification. This work was performed in a well-ventilated fume hood due to the corrosive nature of bifluoride.

Synthesis

Synthesis of $K_2[ReF_6]$

$K_2[ReF_6]$ was prepared by melting $K_2[ReBr_6]$ (2 g, 2.69 mmol) with excess KHF_2 (14 g, 0.18 mol) in a nickel crucible at 400 ^{O}C for 30 minutes in a box furnace. The resulting greyish-solid product formed was allowed to cool to room temperature and was washed first with MeOH (4 x 10 mL). Subsequently, the product was washed with several aliquots of H_2O-MeOH mixture (3 x 5 mL, 1:4 volume ratios) and centrifuged. The pink solid obtained was dissolved in warm water (5-10 mL, 80 ^{O}C) and evaporated slowly at room temperature. The resulted pink crystal of $K_2[ReF_6]$ was recrystallized from warm water (5 mL, 80 ^{O}C) and colorless crystals of $K_2[ReF_6]$ were obtained. Yield: 661 mg, 1.7 mmol (65%). IR (KBr, cm^{-1}): 518, 484 sh (Re─F). Raman (cm^{-1}): 624, 539, 244, 224 (Re─F).

Syntheses of $A_2[ReF_6]$ (A = Rb, Cs,) salts

$K_2[ReF_6]$ (151 mg, 0.4 mmol) was dissolved in 4 mL of warm H_2O (80 ^{O}C). MF (M = Rb, Cs) (0.8 mmol) dissolved 1 mL of hot H_2O (80 ^{O}C) was added dropwise. The solution was allowed to evaporate slowly at room temperature. Crystals of $Rb_2[ReF_6]$ and $Cs_2[ReF_6]$ were formed in 24 hours and washed first with cold H_2O (3 x 2 mL) to remove other fluoride impurities followed by isopropanol (3 x 1 mL), and diethyl ether (3 x 1 mL). $Rb_2[ReF_6]$ yield: 156 mg, 0.33 mmol (83%). IR (KBr, cm^{-1}): 521 (Re─F). Raman (cm^{-1}): 616, 535, 232, 222 (Re─F). $Cs_2[ReF_6]$ yield:

175 mg, 0.276 mmol (77%). IR (KBr, cm^{-1}): 507, 480 sh (Re─F). Raman (cm^{-1}): 609, 529, 218 (Re─F).

Physical Measurements

Vibrational Spectra. IR spectra were measured on a Shimadzu IR Affinity-1 spectrometer between 400 and 4000 cm^{-1}. Raman spectra were recorded on a HORIBA T64000 triple spectrometer operating at 30 mW in subtractive mode. The spectra were taken from pure single crystals at room temperature using a 514.5 nm (Kr/Ar) laser line.

Single Crystal XRD Measurement. Crystals were mounted under Paratone oil on a glass fiber. The data were collected on a Bruker Apex II equipment with an Oxford nitrogen cryostream system operating at 100 K. Structural solution and refinement were performed using APEX3 v2016.5-0.

Geometry.

Anisotropic displacement parameters [Å^2] for K_2ReF_6						
Atom	U_{11}	U_{22}	U_{33}	U_{12}	U_{13}	U_{23}
Re1	0.00883(16)	0.00883(16)	0.0082(2)	0.00441(8)	0.00000	0.00000
F1	0.0119(13)	0.0162(10)	0.0117(12)	0.0060(6)	-0.0012(10)	-0.0006(5)
K1	0.0118(5)	0.0118(5)	0.0096(6)	0.0059(2)	0.00000	0.00000

Geometric parameters, bond lengths [Å] and angles [°] for K_2ReF_6			
Re1—F1	1.948(3)	F1—K1^{viii}	2.9325(10)
Re1—F1^{i}	1.948(3)	F1—K1^{vi}	2.946(3)
Re1—F1^{ii}	1.948(3)	K1—F1^{xi}	2.762(3)
Re1—F1^{iii}	1.948(3)	K1—F1^{x}	2.762(3)
Re1—F1^{iv}	1.948(3)	K1—F1^{xii}	2.762(3)
Re1—F1^{v}	1.948(3)	K1—F1^{xiii}	2.9325(11)
Re1—K1^{i}	3.6263(13)	K1—F1^{xiv}	2.9325(10)
Re1—K1^{vi}	3.6263(13)	K1—F1^{iv}	2.9325(11)
Re1—K1^{vii}	3.6263(13)	K1—F1^{xv}	2.9325(11)
Re1—K1	3.6263(13)	K1—F1^{xvi}	2.9325(11)
Re1—K1^{viii}	3.6263(13)	K1—F1^{xvii}	2.946(3)
Re1—K1^{ix}	3.6263(13)	K1—F1^{ii}	2.946(3)
F1—K1^{x}	2.762(3)	K1—F1^{vi}	2.946(3)
F1—K1	2.9325(11)		
F1—Re1—F1^{i}	180	K1—F1—K1^{viii}	168.22(13)
F1—Re1—F1^{ii}	86.08(12)	Re1—F1—K1^{vi}	93.38(11)

$F1^{i}$—Re1—$F1^{ii}$	93.92(12)	$K1^{x}$—F1—$K1^{vi}$	105.55(10)
F1—Re1—$F1^{iii}$	93.92(12)	K1—F1—$K1^{vi}$	94.27(6)
$F1^{i}$—Re1—$F1^{iii}$	86.08(12)	$K1^{viii}$—F1—$K1^{vi}$	94.27(6)
$F1^{ii}$—Re1—$F1^{iii}$	180.00(19)	$F1^{xi}$—K1—$F1^{x}$	65.46(11)
F1—Re1—$F1^{iv}$	93.92(12)	$F1^{xi}$—K1—$F1^{xii}$	65.46(11)
$F1^{i}$—Re1—$F1^{iv}$	86.08(12)	$F1^{x}$—K1—$F1^{xii}$	65.46(11)
$F1^{ii}$—Re1—$F1^{iv}$	86.08(12)	$F1^{xi}$—K1—$F1^{xiii}$	62.44(10)
$F1^{iii}$—Re1—$F1^{iv}$	93.92(12)	$F1^{x}$—K1—$F1^{xiii}$	127.81(6)
F1—Re1—$F1^{v}$	86.08(12)	$F1^{xii}$—K1—$F1^{xiii}$	95.05(6)
$F1^{i}$—Re1—$F1^{v}$	93.92(12)	$F1^{xi}$—K1—$F1^{xiv}$	62.44(10)
$F1^{ii}$—Re1—$F1^{v}$	93.92(12)	$F1^{x}$—K1—$F1^{xiv}$	95.05(6)
$F1^{iii}$—Re1—$F1^{v}$	86.08(12)	$F1^{xii}$—K1—$F1^{xiv}$	127.81(6)
$F1^{iv}$—Re1—$F1^{v}$	180.00(7)	$F1^{xiii}$—K1—$F1^{xiv}$	58.10(11)
F1—Re1—$K1^{i}$	126.206(14)	$F1^{xi}$—K1—$F1^{iv}$	95.05(6)
$F1^{i}$—Re1—$K1^{i}$	53.794(14)	$F1^{x}$—K1—$F1^{iv}$	127.81(6)
$F1^{ii}$—Re1—$K1^{i}$	125.81(9)	$F1^{xii}$—K1—$F1^{iv}$	62.44(10)
$F1^{iii}$—Re1—$K1^{i}$	54.19(9)	$F1^{xiii}$—K1—$F1^{iv}$	61.22(12)
$F1^{iv}$—Re1—$K1^{i}$	126.205(14)	$F1^{xiv}$—K1—$F1^{iv}$	118.98(2)
$F1^{v}$—Re1—$K1^{i}$	53.795(14)	$F1^{xi}$—K1—$F1^{xv}$	95.05(6)
F1—Re1—$K1^{vi}$	54.19(9)	$F1^{x}$—K1—$F1^{xv}$	62.44(10)
$F1^{i}$—Re1—$K1^{vi}$	125.81(9)	$F1^{xii}$—K1—$F1^{xv}$	127.81(6)
$F1^{ii}$—Re1—$K1^{vi}$	53.794(14)	$F1^{xiii}$—K1—$F1^{xv}$	118.98(2)
$F1^{iii}$—Re1—$K1^{vi}$	126.206(14)	$F1^{xiv}$—K1—$F1^{xv}$	61.22(12)
$F1^{iv}$—Re1—$K1^{vi}$	126.205(14)	$F1^{iv}$—K1—$F1^{xv}$	168.22(13)
$F1^{v}$—Re1—$K1^{vi}$	53.795(14)	$F1^{xi}$—K1—$F1^{xvi}$	127.81(6)
$K1^{i}$—Re1—$K1^{vi}$	107.11(3)	$F1^{x}$—K1—$F1^{xvi}$	62.44(10)
F1—Re1—$K1^{vii}$	125.81(9)	$F1^{xii}$—K1—$F1^{xvi}$	95.05(6)
$F1^{i}$—Re1—$K1^{vii}$	54.19(9)	$F1^{xiii}$—K1—$F1^{xvi}$	168.22(13)
$F1^{ii}$—Re1—$K1^{vii}$	126.206(14)	$F1^{xiv}$—K1—$F1^{xvi}$	118.98(2)
$F1^{iii}$—Re1—$K1^{vii}$	53.794(14)	$F1^{iv}$—K1—$F1^{xvi}$	118.98(2)
$F1^{iv}$—Re1—$K1^{vii}$	53.795(14)	$F1^{xv}$—K1—$F1^{xvi}$	58.10(11)
$F1^{v}$—Re1—$K1^{vii}$	126.205(14)	$F1^{xi}$—K1—F1	127.81(6)
$K1^{i}$—Re1—$K1^{vii}$	72.89(3)	$F1^{x}$—K1—F1	95.05(6)
$K1^{vi}$—Re1—$K1^{vii}$	180	$F1^{xii}$—K1—F1	62.44(10)
F1—Re1—K1	53.794(14)	$F1^{xiii}$—K1—F1	118.98(2)
$F1^{i}$—Re1—K1	126.206(14)	$F1^{xiv}$—K1—F1	168.22(13)
$F1^{ii}$—Re1—K1	54.19(9)	$F1^{iv}$—K1—F1	58.09(11)
$F1^{iii}$—Re1—K1	125.81(9)	$F1^{xv}$—K1—F1	118.98(2)
$F1^{iv}$—Re1—K1	53.794(14)	$F1^{xvi}$—K1—F1	61.22(11)
$F1^{v}$—Re1—K1	126.206(14)	$F1^{xi}$—K1—$F1^{xvii}$	105.55(10)
$K1^{i}$—Re1—K1	180	$F1^{x}$—K1—$F1^{xvii}$	144.70(4)
$K1^{vi}$—Re1—K1	72.89(3)	$F1^{xii}$—K1—$F1^{xvii}$	144.70(4)
$K1^{vii}$—Re1—K1	107.11(3)	$F1^{xiii}$—K1—$F1^{xvii}$	53.8(1)
F1—Re1—$K1^{viii}$	53.795(14)	$F1^{xiv}$—K1—$F1^{xvii}$	53.8(1)
$F1^{i}$—Re1—$K1^{viii}$	126.205(14)	$F1^{iv}$—K1—$F1^{xvii}$	85.73(6)

F1^{ii}—Re1—K1^{viii}	126.206(14)	F1^{xv}—K1—F1^{xvii}	85.73(6)
F1^{iii}—Re1—K1^{viii}	53.794(14)	F1^{xvi}—K1—F1^{xvii}	114.69(6)
F1^{iv}—Re1—K1^{viii}	125.81(9)	F1—K1—F1^{xvii}	114.69(6)
F1^{v}—Re1—K1^{viii}	54.19(9)	F1^{xi}—K1—F1^{ii}	144.70(4)
K1^{i}—Re1—K1^{viii}	72.90(3)	F1^{x}—K1—F1^{ii}	144.70(4)
K1^{vi}—Re1—K1^{viii}	72.90(3)	F1^{xii}—K1—F1^{ii}	105.55(10)
K1^{vii}—Re1—K1^{viii}	107.10(3)	F1^{xiii}—K1—F1^{ii}	85.73(6)
K1—Re1—K1^{viii}	107.10(3)	F1^{xiv}—K1—F1^{ii}	114.69(6)
F1—Re1—K1^{ix}	126.205(14)	F1^{iv}—K1—F1^{ii}	53.8(1)
F1^{i}—Re1—K1^{ix}	53.795(14)	F1^{xv}—K1—F1^{ii}	114.69(6)
F1^{ii}—Re1—K1^{ix}	53.794(14)	F1^{xvi}—K1—F1^{ii}	85.73(6)
F1^{iii}—Re1—K1^{ix}	126.206(14)	F1—K1—F1^{ii}	53.8(1)
F1^{iv}—Re1—K1^{ix}	54.19(9)	F1^{xvii}—K1—F1^{ii}	60.91(10)
F1^{v}—Re1—K1^{ix}	125.81(9)	F1^{xi}—K1—F1^{vi}	144.70(4)
K1^{i}—Re1—K1^{ix}	107.10(3)	F1^{x}—K1—F1^{vi}	105.55(10)
K1^{vi}—Re1—K1^{ix}	107.10(3)	F1^{xii}—K1—F1^{vi}	144.70(4)
K1^{vii}—Re1—K1^{ix}	72.90(3)	F1^{xiii}—K1—F1^{vi}	114.69(6)
K1—Re1—K1^{ix}	72.90(3)	F1^{xiv}—K1—F1^{vi}	85.73(6)
K1^{viii}—Re1—K1^{ix}	180	F1^{iv}—K1—F1^{vi}	114.69(6)
Re1—F1—K1^{x}	161.08(14)	F1^{xv}—K1—F1^{vi}	53.8(1)
Re1—F1—K1	93.79(6)	F1^{xvi}—K1—F1^{vi}	53.8(1)
K1^{x}—F1—K1	84.95(6)	F1—K1—F1^{vi}	85.73(6)
Re1—F1—K1^{viii}	93.79(6)	F1^{xvii}—K1—F1^{vi}	60.91(10)
K1^{x}—F1—K1^{viii}	84.95(6)	F1^{ii}—K1—F1^{vi}	60.91(10)

Symmetry codes: (i) -x, -y, -z; (ii) x-y, x, -z; (iii) -x+y, -x, z; (iv) -y, x-y, z; (v) y, -x+y, -z; (vi) 1-x, 1-y, -z; (vii) -1+x, -1+y, z; (viii) x, -1+y, z; (ix) -x, 1-y, -z; (x) 1-x, 1-y, 1-z; (xi) y, 1-x+y, 1-z; (xii) x-y, x, 1-z; (xiii) -x+y, 1-x, z; (xiv) x, 1+y, z; (xv) 1-y, 1+x-y, z; (xvi) 1-x+y, 1-x, z; (xvii) y, 1-x+y, -z.

Geometry.

Anisotropic displacement parameters [Å^2] for Rb_2ReF_6						
Atom	U_{11}	U_{22}	U_{33}	U_{12}	U_{13}	U_{23}
Re1	0.0165(13)	0.0165(13)	0.0183(15)	0.0082(6)	0.00000	0.00000
Rb1	0.0160(15)	0.0160(15)	0.0148(17)	0.0080(8)	0.00000	0.00000
F1	0.014(4)	0.017(4)	0.015(6)	0.007(2)	-0.003(4)	-0.0013(18)

Geometric parameters, bond lengths [Å] and angles [°] for Rb_2ReF_6			
Re1—F1^{i}	1.944(9)	Rb1—F1^{xiii}	2.912(8)
Re1—F1^{ii}	1.944(9)	Rb1—F1^{xiv}	3.0208(17)
Re1—F1^{iii}	1.944(9)	Rb1—F1^{xi}	3.0207(17)

Re1—F1^{iv}	1.944(9)	Rb1—F1^{xv}	3.0208(17)
Re1—F1^{v}	1.944(9)	Rb1—F1^{xvi}	3.0208(17)
Re1—F1	1.944(9)	Rb1—F1^{vii}	3.0208(17)
Re1—Rb1^{vi}	3.736(2)	Rb1—F1^{xvii}	3.0208(17)
Re1—Rb1^{vii}	3.736(2)	Rb1—F1^{xviii}	3.068(9)
Re1—Rb1^{viii}	3.736(2)	Rb1—F1^{xix}	3.068(9)
Re1—Rb1^{ix}	3.736(2)	Rb1—F1^{xx}	3.068(9)
Re1—Rb1^{x}	3.736(2)	F1—Rb1^{vii}	3.0207(17)
Re1—Rb1^{xi}	3.736(2)	F1—Rb1^{xi}	3.0208(17)
Rb1—F1	2.912(8)	F1—Rb1^{ix}	3.068(9)
Rb1—F1^{xii}	2.912(8)		
F1^{i}—Re1—F1^{ii}	93.2(3)	F1^{xiii}—Rb1—F1^{xiv}	96.30(16)
F1^{i}—Re1—F1^{iii}	86.8(3)	F1—Rb1—F1^{xi}	96.30(16)
F1^{ii}—Re1—F1^{iii}	180.000	F1^{xii}—Rb1—F1^{xi}	62.4(3)
F1^{i}—Re1—F1^{iv}	93.2(3)	F1^{xiii}—Rb1—F1^{xi}	128.26(12)
F1^{ii}—Re1—F1^{iv}	93.2(3)	F1^{xiv}—Rb1—F1^{xi}	55.7(4)
F1^{iii}—Re1—F1^{iv}	86.8(3)	F1—Rb1—F1^{xv}	128.26(12)
F1^{i}—Re1—F1^{v}	86.8(3)	F1^{xii}—Rb1—F1^{xv}	96.30(16)
F1^{ii}—Re1—F1^{v}	86.8(3)	F1^{xiii}—Rb1—F1^{xv}	62.4(3)
F1^{iii}—Re1—F1^{v}	93.2(3)	F1^{xiv}—Rb1—F1^{xv}	63.4(4)
F1^{iv}—Re1—F1^{v}	180.000	F1^{xi}—Rb1—F1^{xv}	118.72(6)
F1^{i}—Re1—F1	180.0(6)	F1—Rb1—F1^{xvi}	62.4(3)
F1^{ii}—Re1—F1	86.8(3)	F1^{xii}—Rb1—F1^{xvi}	96.30(16)
F1^{iii}—Re1—F1	93.2(3)	F1^{xiii}—Rb1—F1^{xvi}	128.26(12)
F1^{iv}—Re1—F1	86.8(3)	F1^{xiv}—Rb1—F1^{xvi}	118.72(6)
F1^{v}—Re1—F1	93.2(3)	F1^{xi}—Rb1—F1^{xvi}	63.4(4)
F1^{i}—Re1—Rb1^{vi}	53.63(3)	F1^{xv}—Rb1—F1^{xvi}	166.4(3)
F1^{ii}—Re1—Rb1^{vi}	53.63(3)	F1—Rb1—F1^{vii}	96.30(16)
F1^{iii}—Re1—Rb1^{vi}	126.37(3)	F1^{xii}—Rb1—F1^{vii}	128.26(12)
F1^{iv}—Re1—Rb1^{vi}	125.0(2)	F1^{xiii}—Rb1—F1^{vii}	62.4(3)
F1^{v}—Re1—Rb1^{vi}	55.0(2)	F1^{xiv}—Rb1—F1^{vii}	118.72(6)
F1—Re1—Rb1^{vi}	126.37(3)	F1^{xi}—Rb1—F1^{vii}	166.4(3)
F1^{i}—Re1—Rb1^{vii}	126.37(3)	F1^{xv}—Rb1—F1^{vii}	55.7(4)
F1^{ii}—Re1—Rb1^{vii}	126.37(3)	F1^{xvi}—Rb1—F1^{vii}	118.72(6)
F1^{iii}—Re1—Rb1^{vii}	53.63(3)	F1—Rb1—F1^{xvii}	62.4(3)
F1^{iv}—Re1—Rb1^{vii}	55.0(2)	F1^{xii}—Rb1—F1^{xvii}	128.26(12)
F1^{v}—Re1—Rb1^{vii}	125.0(2)	F1^{xiii}—Rb1—F1^{xvii}	96.30(16)
F1—Re1—Rb1^{vii}	53.63(3)	F1^{xiv}—Rb1—F1^{xvii}	166.4(3)
Rb1^{vi}—Re1—Rb1^{vii}	180.000	F1^{xi}—Rb1—F1^{xvii}	118.72(6)
F1^{i}—Re1—Rb1^{viii}	55.0(2)	F1^{xv}—Rb1—F1^{xvii}	118.72(6)
F1^{ii}—Re1—Rb1^{viii}	126.37(3)	F1^{xvi}—Rb1—F1^{xvii}	55.7(4)
F1^{iii}—Re1—Rb1^{viii}	53.63(3)	F1^{vii}—Rb1—F1^{xvii}	63.4(4)
F1^{iv}—Re1—Rb1^{viii}	126.37(3)	F1—Rb1—F1^{xviii}	144.21(12)
F1^{v}—Re1—Rb1^{viii}	53.63(3)	F1^{xii}—Rb1—F1^{xviii}	104.3(3)
F1—Re1—Rb1^{viii}	125.0(2)	F1^{xiii}—Rb1—F1^{xviii}	144.21(12)

$Rb1^{vi}—Re1—Rb1^{viii}$	73.22(6)	$F1^{xiv}—Rb1—F1^{xviii}$	52.0(3)
$Rb1^{vii}—Re1—Rb1^{viii}$	106.78(6)	$F1^{xi}—Rb1—F1^{xviii}$	52.0(3)
$F1^{i}—Re1—Rb1^{ix}$	125.0(2)	$F1^{xv}—Rb1—F1^{xviii}$	85.90(18)
$F1^{ii}—Re1—Rb1^{ix}$	53.63(3)	$F1^{xvi}—Rb1—F1^{xviii}$	85.90(18)
$F1^{iii}—Re1—Rb1^{ix}$	126.37(3)	$F1^{vii}—Rb1—F1^{xviii}$	114.35(13)
$F1^{iv}—Re1—Rb1^{ix}$	53.63(3)	$F1^{xvii}—Rb1—F1^{xviii}$	114.35(13)
$F1^{v}—Re1—Rb1^{ix}$	126.37(3)	$F1—Rb1—F1^{xix}$	144.21(12)
$F1—Re1—Rb1^{ix}$	55.0(2)	$F1^{xii}—Rb1—F1^{xix}$	144.21(12)
$Rb1^{vi}—Re1—Rb1^{ix}$	106.78(6)	$F1^{xiii}—Rb1—F1^{xix}$	104.3(3)
$Rb1^{vii}—Re1—Rb1^{ix}$	73.22(6)	$F1^{xiv}—Rb1—F1^{xix}$	85.90(18)
$Rb1^{viii}—Re1—Rb1^{ix}$	180.000	$F1^{xi}—Rb1—F1^{xix}$	114.35(13)
$F1^{i}—Re1—Rb1^{x}$	53.63(3)	$F1^{xv}—Rb1—F1^{xix}$	52.0(3)
$F1^{ii}—Re1—Rb1^{x}$	125.0(2)	$F1^{xvi}—Rb1—F1^{xix}$	114.35(13)
$F1^{iii}—Re1—Rb1^{x}$	55.0(2)	$F1^{vii}—Rb1—F1^{xix}$	52.0(3)
$F1^{iv}—Re1—Rb1^{x}$	53.63(3)	$F1^{xvii}—Rb1—F1^{xix}$	85.90(18)
$F1^{v}—Re1—Rb1^{x}$	126.37(3)	$F1^{xviii}—Rb1—F1^{xix}$	62.3(3)
$F1—Re1—Rb1^{x}$	126.37(3)	$F1—Rb1—F1^{xx}$	104.3(3)
$Rb1^{vi}—Re1—Rb1^{x}$	106.78(6)	$F1^{xii}—Rb1—F1^{xx}$	144.21(12)
$Rb1^{vii}—Re1—Rb1^{x}$	73.22(6)	$F1^{xiii}—Rb1—F1^{xx}$	144.21(12)
$Rb1^{viii}—Re1—Rb1^{x}$	73.22(6)	$F1^{xiv}—Rb1—F1^{xx}$	114.35(13)
$Rb1^{ix}—Re1—Rb1^{x}$	106.78(6)	$F1^{xi}—Rb1—F1^{xx}$	85.90(18)
$F1^{i}—Re1—Rb1^{xi}$	126.37(3)	$F1^{xv}—Rb1—F1^{xx}$	114.35(13)
$F1^{ii}—Re1—Rb1^{xi}$	55.0(2)	$F1^{xvi}—Rb1—F1^{xx}$	52.0(3)
$F1^{iii}—Re1—Rb1^{xi}$	125.0(2)	$F1^{vii}—Rb1—F1^{xx}$	85.90(18)
$F1^{iv}—Re1—Rb1^{xi}$	126.37(3)	$F1^{xvii}—Rb1—F1^{xx}$	52.0(3)
$F1^{v}—Re1—Rb1^{xi}$	53.63(3)	$F1^{xviii}—Rb1—F1^{xx}$	62.3(3)
$F1—Re1—Rb1^{xi}$	53.63(3)	$F1^{xix}—Rb1—F1^{xx}$	62.3(3)
$Rb1^{vi}—Re1—Rb1^{xi}$	73.22(6)	Re1—F1—Rb1	162.0(4)
$Rb1^{vii}—Re1—Rb1^{xi}$	106.78(6)	$Re1—F1—Rb1^{vii}$	95.17(17)
$Rb1^{viii}—Re1—Rb1^{xi}$	106.78(6)	$Rb1—F1—Rb1^{vii}$	83.70(16)
$Rb1^{ix}—Re1—Rb1^{xi}$	73.22(6)	$Re1—F1—Rb1^{xi}$	95.17(17)
$Rb1^{x}—Re1—Rb1^{xi}$	180.000	$Rb1—F1—Rb1^{xi}$	83.70(16)
$F1—Rb1—F1^{xii}$	66.1(3)	$Rb1^{vii}—F1—Rb1^{xi}$	166.3(3)
$F1—Rb1—F1^{xiii}$	66.1(3)	$Re1—F1—Rb1^{ix}$	93.7(3)
$F1^{xii}—Rb1—F1^{xiii}$	66.1(3)	$Rb1—F1—Rb1^{ix}$	104.3(3)
$F1—Rb1—F1^{xiv}$	128.26(12)	$Rb1^{vii}—F1—Rb1^{ix}$	94.10(18)
$F1^{xii}—Rb1—F1^{xiv}$	62.4(3)	$Rb1^{xi}—F1—Rb1^{ix}$	94.10(18)

Symmetry codes: (i) 2-x, 2-y, 2-z; (ii) y, 1-x+y, 2-z; (iii) 2-y, 1+x-y, z; (iv) 1+x-y, x, 2-z; (v) 1-x+y, 2-x, z; (vi) 1+x, 1+y, 1+z; (vii) 1-x, 1-y, 1-z; (viii) 2-x, 2-y, 1-z; (ix) x, y, 1+z; (x) 1+x, y, 1+z; (xi) 1-x, 2-y, 1-z; (xii) 1-y, 1+x-y, z; (xiii) -x+y, 1-x, z; (xiv) x-y, x, 1-z; (xv) -1+y, -x+y, 1-z; (xvi) y, 1-x+y, 1-z; (xvii) 1+x-y, x, 1-z; (xviii) 1-y, 1+x-y, -1+z; (xix) -x+y, 1-x, -1+z; (xx) x, y, -1+z.

Geometry.

Anisotropic displacement parameters [Å^2] for Cs_2ReF_6						
Atom	U_{11}	U_{22}	U_{33}	U_{12}	U_{13}	U_{23}
Re1	0.00484(16)	0.00484(16)	0.0032(2)	0.00242(8)	0.00000	0.00000
Cs1	0.00664(16)	0.00664(16)	0.0053(2)	0.00332(8)	0.00000	0.00000
F1	0.0088(8)	0.0107(6)	0.0075(9)	0.0044(4)	-0.0024(6)	-0.0012(3)

Geometric parameters, bond lengths [Å] and angles [°] for Cs_2ReF_6			
Re1—F1^{i}	1.9595(18)	Cs1—F1^{xiii}	3.0953(18)
Re1—F1^{ii}	1.9595(18)	Cs1—F1^{xiv}	3.1655(5)
Re1—F1^{iii}	1.9595(18)	Cs1—F1^{x}	3.1655(6)
Re1—F1^{iv}	1.9595(18)	Cs1—F1^{xv}	3.1655(6)
Re1—F1^{v}	1.9595(18)	Cs1—F1^{xvi}	3.1655(6)
Re1—F1	1.9595(18)	Cs1—F1^{vii}	3.1655(6)
Re1—Cs1^{vi}	3.9100(6)	Cs1—F1^{xvii}	3.1655(6)
Re1—Cs1^{vii}	3.9100(6)	Cs1—F1^{xviii}	3.2244(19)
Re1—Cs1^{viii}	3.9100(6)	Cs1—F1^{xix}	3.2244(19)
Re1—Cs1^{ix}	3.9100(6)	Cs1—F1^{xx}	3.2244(19)
Re1—Cs1^{x}	3.9101(6)	F1—Cs1^{vii}	3.1654(6)
Re1—Cs1^{xi}	3.9101(6)	F1—Cs1^{x}	3.1655(6)
Cs1—F1	3.0953(18)	F1—Cs1^{viii}	3.2244(19)
Cs1—F1^{xii}	3.0953(18)		
F1^{i}—Re1—F1^{ii}	93.14(7)	F1^{xiii}—Cs1—F1^{xiv}	97.70(3)
F1^{i}—Re1—F1^{iii}	86.86(7)	F1—Cs1—F1^{x}	97.70(3)
F1^{ii}—Re1—F1^{iii}	180.000	F1^{xii}—Cs1—F1^{x}	62.38(6)
F1^{i}—Re1—F1^{iv}	93.14(7)	F1^{xiii}—Cs1—F1^{x}	129.121(17)
F1^{ii}—Re1—F1^{iv}	93.14(7)	F1^{xiv}—Cs1—F1^{x}	53.43(7)
F1^{iii}—Re1—F1^{iv}	86.86(7)	F1—Cs1—F1^{xv}	129.121(17)
F1^{i}—Re1—F1^{v}	86.86(7)	F1^{xii}—Cs1—F1^{xv}	97.70(3)
F1^{ii}—Re1—F1^{v}	86.86(7)	F1^{xiii}—Cs1—F1^{xv}	62.38(6)
F1^{iii}—Re1—F1^{v}	93.14(7)	F1^{xiv}—Cs1—F1^{xv}	65.44(7)
F1^{iv}—Re1—F1^{v}	180.000	F1^{x}—Cs1—F1^{xv}	118.324(15)
F1^{i}—Re1—F1	180.00(8)	F1—Cs1—F1^{xvi}	62.38(6)
F1^{ii}—Re1—F1	86.86(7)	F1^{xii}—Cs1—F1^{xvi}	97.70(3)
F1^{iii}—Re1—F1	93.14(7)	F1^{xiii}—Cs1—F1^{xvi}	129.121(17)
F1^{iv}—Re1—F1	86.86(7)	F1^{xiv}—Cs1—F1^{xvi}	118.324(14)
F1^{v}—Re1—F1	93.14(7)	F1^{x}—Cs1—F1^{xvi}	65.44(7)
F1^{i}—Re1—Cs1^{vi}	53.532(6)	F1^{xv}—Cs1—F1^{xvi}	163.83(6)
F1^{ii}—Re1—Cs1^{vi}	124.74(6)	F1—Cs1—F1^{vii}	97.70(3)
F1^{iii}—Re1—Cs1^{vi}	55.27(6)	F1^{xii}—Cs1—F1^{vii}	129.121(17)
F1^{iv}—Re1—Cs1^{vi}	53.533(6)	F1^{xiii}—Cs1—F1^{vii}	62.38(6)

$F1^{v}$—Re1—$Cs1^{vi}$	126.467(6)	$F1^{xiv}$—Cs1—$F1^{vii}$	118.324(15)
F1—Re1—$Cs1^{vi}$	126.468(6)	$F1^{x}$—Cs1—$F1^{vii}$	163.83(6)
$F1^{i}$—Re1—$Cs1^{vii}$	126.468(6)	$F1^{xv}$—Cs1—$F1^{vii}$	53.43(7)
$F1^{ii}$—Re1—$Cs1^{vii}$	55.26(6)	$F1^{xvi}$—Cs1—$F1^{vii}$	118.324(15)
$F1^{iii}$—Re1—$Cs1^{vii}$	124.74(6)	F1—Cs1—$F1^{xvii}$	62.38(6)
$F1^{iv}$—Re1—$Cs1^{vii}$	126.467(7)	$F1^{xii}$—Cs1—$F1^{xvii}$	129.121(17)
$F1^{v}$—Re1—$Cs1^{vii}$	53.533(6)	$F1^{xiii}$—Cs1—$F1^{xvii}$	97.70(3)
F1—Re1—$Cs1^{vii}$	53.532(6)	$F1^{xiv}$—Cs1—$F1^{xvii}$	163.83(6)
$Cs1^{vi}$—Re1—$Cs1^{vii}$	180.000	$F1^{x}$—Cs1—$F1^{xvii}$	118.324(15)
$F1^{i}$—Re1—$Cs1^{viii}$	124.74(6)	$F1^{xv}$—Cs1—$F1^{xvii}$	118.324(14)
$F1^{ii}$—Re1—$Cs1^{viii}$	53.532(6)	$F1^{xvi}$—Cs1—$F1^{xvii}$	53.43(7)
$F1^{iii}$—Re1—$Cs1^{viii}$	126.468(6)	$F1^{vii}$—Cs1—$F1^{xvii}$	65.44(7)
$F1^{iv}$—Re1—$Cs1^{viii}$	53.533(6)	F1—Cs1—$F1^{xviii}$	143.51(2)
$F1^{v}$—Re1—$Cs1^{viii}$	126.467(6)	$F1^{xii}$—Cs1—$F1^{xviii}$	102.55(5)
F1—Re1—$Cs1^{viii}$	55.26(6)	$F1^{xiii}$—Cs1—$F1^{xviii}$	143.51(2)
$Cs1^{vi}$—Re1—$Cs1^{viii}$	106.553(9)	$F1^{xiv}$—Cs1—$F1^{xviii}$	49.87(5)
$Cs1^{vii}$—Re1—$Cs1^{viii}$	73.447(9)	$F1^{x}$—Cs1—$F1^{xviii}$	49.87(5)
$F1^{i}$—Re1—$Cs1^{ix}$	55.26(6)	$F1^{xv}$—Cs1—$F1^{xviii}$	85.93(3)
$F1^{ii}$—Re1—$Cs1^{ix}$	126.468(6)	$F1^{xvi}$—Cs1—$F1^{xviii}$	85.93(3)
$F1^{iii}$—Re1—$Cs1^{ix}$	53.532(6)	$F1^{vii}$—Cs1—$F1^{xviii}$	113.96(2)
$F1^{iv}$—Re1—$Cs1^{ix}$	126.467(6)	$F1^{xvii}$—Cs1—$F1^{xviii}$	113.96(2)
$F1^{v}$—Re1—$Cs1^{ix}$	53.533(6)	F1—Cs1—$F1^{xix}$	143.51(2)
F1—Re1—$Cs1^{ix}$	124.74(6)	$F1^{xii}$—Cs1—$F1^{xix}$	143.51(2)
$Cs1^{vi}$—Re1—$Cs1^{ix}$	73.447(9)	$F1^{xiii}$—Cs1—$F1^{xix}$	102.55(5)
$Cs1^{vii}$—Re1—$Cs1^{ix}$	106.553(9)	$F1^{xiv}$—Cs1—$F1^{xix}$	85.93(3)
$Cs1^{viii}$—Re1—$Cs1^{ix}$	180.000	$F1^{x}$—Cs1—$F1^{xix}$	113.96(2)
$F1^{i}$—Re1—$Cs1^{x}$	126.467(6)	$F1^{xv}$—Cs1—$F1^{xix}$	49.87(5)
$F1^{ii}$—Re1—$Cs1^{x}$	126.467(6)	$F1^{xvi}$—Cs1—$F1^{xix}$	113.96(2)
$F1^{iii}$—Re1—$Cs1^{x}$	53.533(6)	$F1^{vii}$—Cs1—$F1^{xix}$	49.87(5)
$F1^{iv}$—Re1—$Cs1^{x}$	55.26(6)	$F1^{xvii}$—Cs1—$F1^{xix}$	85.93(3)
$F1^{v}$—Re1—$Cs1^{x}$	124.74(6)	$F1^{xviii}$—Cs1—$F1^{xix}$	64.10(5)
F1—Re1—$Cs1^{x}$	53.533(6)	F1—Cs1—$F1^{xx}$	102.55(5)
$Cs1^{vi}$—Re1—$Cs1^{x}$	73.448(9)	$F1^{xii}$—Cs1—$F1^{xx}$	143.51(2)
$Cs1^{vii}$—Re1—$Cs1^{x}$	106.552(9)	$F1^{xiii}$—Cs1—$F1^{xx}$	143.51(2)
$Cs1^{viii}$—Re1—$Cs1^{x}$	73.448(9)	$F1^{xiv}$—Cs1—$F1^{xx}$	113.96(2)
$Cs1^{ix}$—Re1—$Cs1^{x}$	106.552(9)	$F1^{x}$—Cs1—$F1^{xx}$	85.93(3)
$F1^{i}$—Re1—$Cs1^{xi}$	53.533(6)	$F1^{xv}$—Cs1—$F1^{xx}$	113.96(2)
$F1^{ii}$—Re1—$Cs1^{xi}$	53.533(6)	$F1^{xvi}$—Cs1—$F1^{xx}$	49.87(5)
$F1^{iii}$—Re1—$Cs1^{xi}$	126.467(6)	$F1^{vii}$—Cs1—$F1^{xx}$	85.93(3)
$F1^{iv}$—Re1—$Cs1^{xi}$	124.74(6)	$F1^{xvii}$—Cs1—$F1^{xx}$	49.87(5)
$F1^{v}$—Re1—$Cs1^{xi}$	55.26(6)	$F1^{xviii}$—Cs1—$F1^{xx}$	64.10(5)
F1—Re1—$Cs1^{xi}$	126.467(6)	$F1^{xix}$—Cs1—$F1^{xx}$	64.10(5)
$Cs1^{vi}$—Re1—$Cs1^{xi}$	106.552(9)	Re1—F1—Cs1	162.68(9)
$Cs1^{vii}$—Re1—$Cs1^{xi}$	73.448(9)	Re1—F1—$Cs1^{vii}$	96.61(3)
$Cs1^{viii}$—Re1—$Cs1^{xi}$	106.552(9)	Cs1—F1—$Cs1^{vii}$	82.30(3)

Cs1^{ix}—Re1—Cs1^{xi}	73.448(9)	Re1—F1—Cs1^{x}	96.61(3)
Cs1^{x}—Re1—Cs1^{xi}	180.000	Cs1—F1—Cs1^{x}	82.30(3)
F1—Cs1—F1^{xii}	67.11(6)	Cs1^{vii}—F1—Cs1^{x}	163.83(6)
F1—Cs1—F1^{xiii}	67.11(6)	Re1—F1—Cs1^{viii}	94.77(7)
F1^{xii}—Cs1—F1^{xiii}	67.11(6)	Cs1—F1—Cs1^{viii}	102.55(5)
F1—Cs1—F1^{xiv}	129.121(17)	Cs1^{vii}—F1—Cs1^{viii}	94.07(3)
F1^{xii}—Cs1—F1^{xiv}	62.38(6)	Cs1^{x}—F1—Cs1^{viii}	94.07(3)

Symmetry codes: (i) 2-x, 2-y, 2-z; (ii) 1+x-y, x, 2-z; (iii) 1-x+y, 2-x, z; (iv) y, 1-x+y, 2-z; (v) 2-y, 1+x-y, z; (vi) 1+x, 1+y, 1+z; (vii) 1-x, 1-y, 1-z; (viii) x, y, 1+z; (ix) 2-x, 2-y, 1-z; (x) 1-x, 2-y, 1-z; (xi) 1+x, y, 1+z; (xii) 1-y, 1+x-y, z; (xiii) -x+y, 1-x, z; (xiv) x-y, x, 1-z; (xv) -1+y, -x+y, 1-z; (xvi) y, 1-x+y, 1-z; (xvii) 1+x-y, x, 1-z; (xviii) 1-y, 1+x-y, -1+z; (xix) -x+y, 1-x, -1+z; (xx) x, y, -1+z.
