# Supplementary material for: Syntheses, Raman spectroscopy and crystal structures of alkali hexa­fluorido­rhenates(IV) revisited
Source: Acta Crystallogr E Crystallogr Commun. 2018 Apr 6;74(Pt 5):646–9. doi: 10.1107/S2056989018005297 (PMC5947479; doi:10.1107/S2056989018005297)
Supplement: Supplementary file 7 [file e-74-00646-sup7.pdf]

Supporting information

Syntheses, Raman spectroscopy and crystal structures of alkali hexafluoridorhenates(IV) – revisited

James Louis-Jean[†], Samundeeswari Mariappan Balasekaran[†], Dean Smith[‡], Ashkan Salamat[‡], Thang Chien Pham[‡], Frederic Poineau[†]

Synopsis

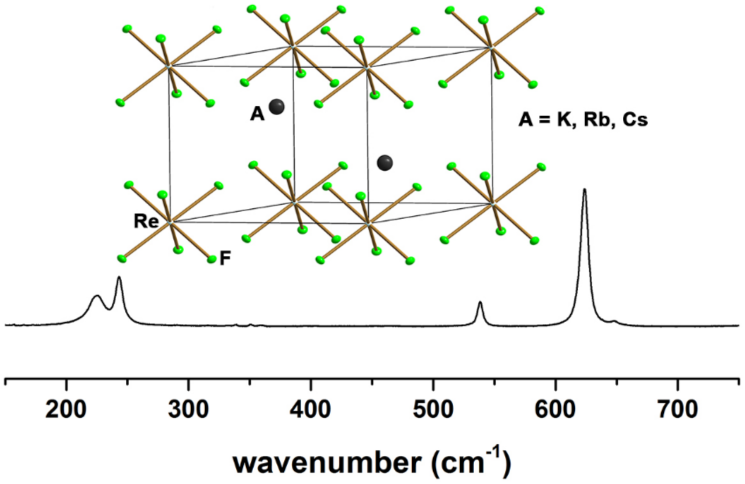

Geometry.

Anisotropic displacement parameters [Å^2] for K_2ReF_6						
Atom	U_{11}	U_{22}	U_{33}	U_{12}	U_{13}	U_{23}
Re1	0.00883(16)	0.00883(16)	0.0082(2)	0.00441(8)	0.00000	0.00000
F1	0.0119(13)	0.0162(10)	0.0117(12)	0.0060(6)	-0.0012(10)	-0.0006(5)
K1	0.0118(5)	0.0118(5)	0.0096(6)	0.0059(2)	0.00000	0.00000

Geometric parameters, bond lengths [Å] and angles [°] for K_2ReF_6			
Re1—F1	1.948(3)	F1—K1^{viii}	2.9325(10)
Re1—F1^{i}	1.948(3)	F1—K1^{vi}	2.946(3)
Re1—F1^{ii}	1.948(3)	K1—F1^{xi}	2.762(3)
Re1—F1^{iii}	1.948(3)	K1—F1^{x}	2.762(3)
Re1—F1^{iv}	1.948(3)	K1—F1^{xii}	2.762(3)
Re1—F1^{v}	1.948(3)	K1—F1^{xiii}	2.9325(11)
Re1—K1^{i}	3.6263(13)	K1—F1^{xiv}	2.9325(10)
Re1—K1^{vi}	3.6263(13)	K1—F1^{iv}	2.9325(11)
Re1—K1^{vii}	3.6263(13)	K1—F1^{xv}	2.9325(11)
Re1—K1	3.6263(13)	K1—F1^{xvi}	2.9325(11)
Re1—K1^{viii}	3.6263(13)	K1—F1^{xvii}	2.946(3)
Re1—K1^{ix}	3.6263(13)	K1—F1^{ii}	2.946(3)
F1—K1^{x}	2.762(3)	K1—F1^{vi}	2.946(3)
F1—K1	2.9325(11)		
F1—Re1—F1^{i}	180.000	K1—F1—K1^{viii}	168.22(13)
F1—Re1—F1^{ii}	86.08(12)	Re1—F1—K1^{vi}	93.38(11)
F1^{i}—Re1—F1^{ii}	93.92(12)	K1^{x}—F1—K1^{vi}	105.55(10)
F1—Re1—F1^{iii}	93.92(12)	K1—F1—K1^{vi}	94.27(6)
F1^{i}—Re1—F1^{iii}	86.08(12)	K1^{viii}—F1—K1^{vi}	94.27(6)
F1^{ii}—Re1—F1^{iii}	180.00(19)	F1^{xi}—K1—F1^{x}	65.46(11)
F1—Re1—F1^{iv}	93.92(12)	F1^{xi}—K1—F1^{xii}	65.46(11)
F1^{i}—Re1—F1^{iv}	86.08(12)	F1^{x}—K1—F1^{xii}	65.46(11)
F1^{ii}—Re1—F1^{iv}	86.08(12)	F1^{xi}—K1—F1^{xiii}	62.44(10)
F1^{iii}—Re1—F1^{iv}	93.92(12)	F1^{x}—K1—F1^{xiii}	127.81(6)
F1—Re1—F1^{v}	86.08(12)	F1^{xii}—K1—F1^{xiii}	95.05(6)
F1^{i}—Re1—F1^{v}	93.92(12)	F1^{xi}—K1—F1^{xiv}	62.44(10)
F1^{ii}—Re1—F1^{v}	93.92(12)	F1^{x}—K1—F1^{xiv}	95.05(6)
F1^{iii}—Re1—F1^{v}	86.08(12)	F1^{xii}—K1—F1^{xiv}	127.81(6)
F1^{iv}—Re1—F1^{v}	180.00(7)	F1^{xiii}—K1—F1^{xiv}	58.10(11)
F1—Re1—K1^{i}	126.206(14)	F1^{xi}—K1—F1^{iv}	95.05(6)
F1^{i}—Re1—K1^{i}	53.794(14)	F1^{x}—K1—F1^{iv}	127.81(6)
F1^{ii}—Re1—K1^{i}	125.81(9)	F1^{xii}—K1—F1^{iv}	62.44(10)
F1^{iii}—Re1—K1^{i}	54.19(9)	F1^{xiii}—K1—F1^{iv}	61.22(12)
F1^{iv}—Re1—K1^{i}	126.205(14)	F1^{xiv}—K1—F1^{iv}	118.98(2)
F1^{v}—Re1—K1^{i}	53.795(14)	F1^{xi}—K1—F1^{xv}	95.05(6)
F1—Re1—K1^{vi}	54.19(9)	F1^{x}—K1—F1^{xv}	62.44(10)
F1^{i}—Re1—K1^{vi}	125.81(9)	F1^{xii}—K1—F1^{xv}	127.81(6)
F1^{ii}—Re1—K1^{vi}	53.794(14)	F1^{xiii}—K1—F1^{xv}	118.98(2)
F1^{iii}—Re1—K1^{vi}	126.206(14)	F1^{xiv}—K1—F1^{xv}	61.22(12)
F1^{iv}—Re1—K1^{vi}	126.205(14)	F1^{iv}—K1—F1^{xv}	168.22(13)
F1^{v}—Re1—K1^{vi}	53.795(14)	F1^{xi}—K1—F1^{xvi}	127.81(6)

$K1^{i}$—Re1—$K1^{vi}$	107.11(3)	$F1^{x}$—K1—$F1^{xvi}$	62.44(10)
F1—Re1—$K1^{vii}$	125.81(9)	$F1^{xii}$—K1—$F1^{xvi}$	95.05(6)
$F1^{i}$—Re1—$K1^{vii}$	54.19(9)	$F1^{xiii}$—K1—$F1^{xvi}$	168.22(13)
$F1^{ii}$—Re1—$K1^{vii}$	126.206(14)	$F1^{xiv}$—K1—$F1^{xvi}$	118.98(2)
$F1^{iii}$—Re1—$K1^{vii}$	53.794(14)	$F1^{iv}$—K1—$F1^{xvi}$	118.98(2)
$F1^{iv}$—Re1—$K1^{vii}$	53.795(14)	$F1^{xv}$—K1—$F1^{xvi}$	58.10(11)
$F1^{v}$—Re1—$K1^{vii}$	126.205(14)	$F1^{xi}$—K1—F1	127.81(6)
$K1^{i}$—Re1—$K1^{vii}$	72.89(3)	$F1^{x}$—K1—F1	95.05(6)
$K1^{vi}$—Re1—$K1^{vii}$	180.000	$F1^{xii}$—K1—F1	62.44(10)
F1—Re1—K1	53.794(14)	$F1^{xiii}$—K1—F1	118.98(2)
$F1^{i}$—Re1—K1	126.206(14)	$F1^{xiv}$—K1—F1	168.22(13)
$F1^{ii}$—Re1—K1	54.19(9)	$F1^{iv}$—K1—F1	58.09(11)
$F1^{iii}$—Re1—K1	125.81(9)	$F1^{xv}$—K1—F1	118.98(2)
$F1^{iv}$—Re1—K1	53.794(14)	$F1^{xvi}$—K1—F1	61.22(11)
$F1^{v}$—Re1—K1	126.206(14)	$F1^{xi}$—K1—$F1^{xvii}$	105.55(10)
$K1^{i}$—Re1—K1	180.000	$F1^{x}$—K1—$F1^{xvii}$	144.70(4)
$K1^{vi}$—Re1—K1	72.89(3)	$F1^{xii}$—K1—$F1^{xvii}$	144.70(4)
$K1^{vii}$—Re1—K1	107.11(3)	$F1^{xiii}$—K1—$F1^{xvii}$	53.8(1)
F1—Re1—$K1^{viii}$	53.795(14)	$F1^{xiv}$—K1—$F1^{xvii}$	53.8(1)
$F1^{i}$—Re1—$K1^{viii}$	126.205(14)	$F1^{iv}$—K1—$F1^{xvii}$	85.73(6)
$F1^{ii}$—Re1—$K1^{viii}$	126.206(14)	$F1^{xv}$—K1—$F1^{xvii}$	85.73(6)
$F1^{iii}$—Re1—$K1^{viii}$	53.794(14)	$F1^{xvi}$—K1—$F1^{xvii}$	114.69(6)
$F1^{iv}$—Re1—$K1^{viii}$	125.81(9)	F1—K1—$F1^{xvii}$	114.69(6)
$F1^{v}$—Re1—$K1^{viii}$	54.19(9)	$F1^{xi}$—K1—$F1^{ii}$	144.70(4)
$K1^{i}$—Re1—$K1^{viii}$	72.90(3)	$F1^{x}$—K1—$F1^{ii}$	144.70(4)
$K1^{vi}$—Re1—$K1^{viii}$	72.90(3)	$F1^{xii}$—K1—$F1^{ii}$	105.55(10)
$K1^{vii}$—Re1—$K1^{viii}$	107.10(3)	$F1^{xiii}$—K1—$F1^{ii}$	85.73(6)
K1—Re1—$K1^{viii}$	107.10(3)	$F1^{xiv}$—K1—$F1^{ii}$	114.69(6)
F1—Re1—$K1^{ix}$	126.205(14)	$F1^{iv}$—K1—$F1^{ii}$	53.8(1)
$F1^{i}$—Re1—$K1^{ix}$	53.795(14)	$F1^{xv}$—K1—$F1^{ii}$	114.69(6)
$F1^{ii}$—Re1—$K1^{ix}$	53.794(14)	$F1^{xvi}$—K1—$F1^{ii}$	85.73(6)
$F1^{iii}$—Re1—$K1^{ix}$	126.206(14)	F1—K1—$F1^{ii}$	53.8(1)
$F1^{iv}$—Re1—$K1^{ix}$	54.19(9)	$F1^{xvii}$—K1—$F1^{ii}$	60.91(10)
$F1^{v}$—Re1—$K1^{ix}$	125.81(9)	$F1^{xi}$—K1—$F1^{vi}$	144.70(4)
$K1^{i}$—Re1—$K1^{ix}$	107.10(3)	$F1^{x}$—K1—$F1^{vi}$	105.55(10)
$K1^{vi}$—Re1—$K1^{ix}$	107.10(3)	$F1^{xii}$—K1—$F1^{vi}$	144.70(4)
$K1^{vii}$—Re1—$K1^{ix}$	72.90(3)	$F1^{xiii}$—K1—$F1^{vi}$	114.69(6)
K1—Re1—$K1^{ix}$	72.90(3)	$F1^{xiv}$—K1—$F1^{vi}$	85.73(6)
$K1^{viii}$—Re1—$K1^{ix}$	180.000	$F1^{iv}$—K1—$F1^{vi}$	114.69(6)
Re1—F1—$K1^{x}$	161.08(14)	$F1^{xv}$—K1—$F1^{vi}$	53.8(1)
Re1—F1—K1	93.79(6)	$F1^{xvi}$—K1—$F1^{vi}$	53.8(1)
$K1^{x}$—F1—K1	84.95(6)	F1—K1—$F1^{vi}$	85.73(6)
Re1—F1—$K1^{viii}$	93.79(6)	$F1^{xvii}$—K1—$F1^{vi}$	60.91(10)
$K1^{x}$—F1—$K1^{viii}$	84.95(6)	$F1^{ii}$—K1—$F1^{vi}$	60.91(10)

Symmetry codes: (i) -x, -y, -z; (ii) x-y, x, -z; (iii) -x+y, -x, z; (iv) -y, x-y, z; (v) y, -x+y, -z; (vi) 1-x, 1-y, -z; (vii) -1+x, -1+y, z; (viii) x, -1+y, z; (ix) -x, 1-y, -z; (x) 1-x, 1-y, 1-z; (xi) y, 1-x+y, 1-z; (xii) x-y, x, 1-z; (xiii) -x+y, 1-x, z; (xiv) x, 1+y, z; (xv) 1-y, 1+x-y, z; (xvi) 1-x+y, 1-x, z; (xvii) y, 1-x+y, -z.

Geometry.

Anisotropic displacement parameters [Å^2] for Rb_2ReF_6						
Atom	U_{11}	U_{22}	U_{33}	U_{12}	U_{13}	U_{23}
Re1	0.0133(6)	0.0133(6)	0.0205(8)	0.0066(3)	0.00000	0.00000
F1	0.015(4)	0.017(3)	0.012(3)	0.0077(19)	0.001(3)	0.0007(14)
Rb1	0.0131(8)	0.0131(8)	0.0152(12)	0.0065(4)	0.00000	0.00000

Geometric parameters, bond lengths [Å] and angles [°] for Rb_2ReF_6			
Re1—F1^{i}	1.945(7)	F1—Rb1^{viii}	3.0181(11)
Re1—F1^{ii}	1.945(7)	F1—Rb1^{vi}	3.058(7)
Re1—F1^{iii}	1.945(7)	Rb1—F1^{xi}	2.907(7)
Re1—F1^{iv}	1.945(7)	Rb1—F1^{x}	2.907(7)
Re1—F1^{v}	1.945(7)	Rb1—F1^{xii}	2.907(7)
Re1—F1	1.945(7)	Rb1—F1^{xiii}	3.0181(11)
Re1—Rb1^{i}	3.733(1)	Rb1—F1^{xiv}	3.0181(11)
Re1—Rb1^{vi}	3.7330(11)	Rb1—F1^{v}	3.0181(11)
Re1—Rb1^{vii}	3.7330(11)	Rb1—F1^{xv}	3.0181(11)
Re1—Rb1	3.733(1)	Rb1—F1^{xvi}	3.0181(11)
Re1—Rb1^{viii}	3.7330(11)	Rb1—F1^{xvii}	3.058(7)
Re1—Rb1^{ix}	3.7330(11)	Rb1—F1^{ii}	3.058(7)
F1—Rb1^{x}	2.907(7)	Rb1—F1^{vi}	3.058(7)
F1—Rb1	3.0181(11)		
F1^{i}—Re1—F1^{ii}	93.5(3)	Rb1—F1—Rb1^{viii}	166.2(3)
F1^{i}—Re1—F1^{iii}	86.5(3)	Re1—F1—Rb1^{vi}	93.9(2)
F1^{ii}—Re1—F1^{iii}	180.0(3)	Rb1^{x}—F1—Rb1^{vi}	104.5(2)
F1^{i}—Re1—F1^{iv}	93.5(3)	Rb1—F1—Rb1^{vi}	94.26(14)
F1^{ii}—Re1—F1^{iv}	93.5(3)	Rb1^{viii}—F1—Rb1^{vi}	94.26(14)
F1^{iii}—Re1—F1^{iv}	86.5(3)	F1^{xi}—Rb1—F1^{x}	65.8(2)
F1^{i}—Re1—F1^{v}	86.5(3)	F1^{xi}—Rb1—F1^{xii}	65.8(2)
F1^{ii}—Re1—F1^{v}	86.5(3)	F1^{x}—Rb1—F1^{xii}	65.8(2)
F1^{iii}—Re1—F1^{v}	93.5(3)	F1^{xi}—Rb1—F1^{xiii}	62.7(2)
F1^{iv}—Re1—F1^{v}	180.000	F1^{x}—Rb1—F1^{xiii}	128.31(8)
F1^{i}—Re1—F1	180.000	F1^{xii}—Rb1—F1^{xiii}	96.30(14)
F1^{ii}—Re1—F1	86.5(3)	F1^{xi}—Rb1—F1^{xiv}	62.7(2)

$F1^{iii}$—Re1—F1	93.5(3)	$F1^{x}$—Rb1—$F1^{xiv}$	96.30(14)
$F1^{iv}$—Re1—F1	86.5(3)	$F1^{xii}$—Rb1—$F1^{xiv}$	128.31(8)
$F1^{v}$—Re1—F1	93.5(3)	$F1^{xiii}$—Rb1—$F1^{xiv}$	56.0(3)
$F1^{i}$—Re1—$Rb1^{i}$	53.64(2)	$F1^{xi}$—Rb1—$F1^{v}$	96.30(14)
$F1^{ii}$—Re1—$Rb1^{i}$	125.2(2)	$F1^{x}$—Rb1—$F1^{v}$	128.31(8)
$F1^{iii}$—Re1—$Rb1^{i}$	54.8(2)	$F1^{xii}$—Rb1—$F1^{v}$	62.7(2)
$F1^{iv}$—Re1—$Rb1^{i}$	53.64(2)	$F1^{xiii}$—Rb1—$F1^{v}$	63.1(3)
$F1^{v}$—Re1—$Rb1^{i}$	126.36(2)	$F1^{xiv}$—Rb1—$F1^{v}$	118.68(6)
F1—Re1—$Rb1^{i}$	126.36(2)	$F1^{xi}$—Rb1—$F1^{xv}$	96.30(14)
$F1^{i}$—Re1—$Rb1^{vi}$	125.2(2)	$F1^{x}$—Rb1—$F1^{xv}$	62.7(2)
$F1^{ii}$—Re1—$Rb1^{vi}$	53.64(2)	$F1^{xii}$—Rb1—$F1^{xv}$	128.31(8)
$F1^{iii}$—Re1—$Rb1^{vi}$	126.36(2)	$F1^{xiii}$—Rb1—$F1^{xv}$	118.68(5)
$F1^{iv}$—Re1—$Rb1^{vi}$	53.64(2)	$F1^{xiv}$—Rb1—$F1^{xv}$	63.1(3)
$F1^{v}$—Re1—$Rb1^{vi}$	126.36(2)	$F1^{v}$—Rb1—$F1^{xv}$	166.2(3)
F1—Re1—$Rb1^{vi}$	54.8(2)	$F1^{xi}$—Rb1—$F1^{xvi}$	128.31(8)
$Rb1^{i}$—Re1—$Rb1^{vi}$	106.77(3)	$F1^{x}$—Rb1—$F1^{xvi}$	62.7(2)
$F1^{i}$—Re1—$Rb1^{vii}$	54.8(2)	$F1^{xii}$—Rb1—$F1^{xvi}$	96.30(14)
$F1^{ii}$—Re1—$Rb1^{vii}$	126.36(2)	$F1^{xiii}$—Rb1—$F1^{xvi}$	166.2(3)
$F1^{iii}$—Re1—$Rb1^{vii}$	53.64(2)	$F1^{xiv}$—Rb1—$F1^{xvi}$	118.68(6)
$F1^{iv}$—Re1—$Rb1^{vii}$	126.36(2)	$F1^{v}$—Rb1—$F1^{xvi}$	118.68(5)
$F1^{v}$—Re1—$Rb1^{vii}$	53.64(2)	$F1^{xv}$—Rb1—$F1^{xvi}$	56.0(3)
F1—Re1—$Rb1^{vii}$	125.2(2)	$F1^{xi}$—Rb1—F1	128.31(8)
$Rb1^{i}$—Re1—$Rb1^{vii}$	73.23(3)	$F1^{x}$—Rb1—F1	96.30(14)
$Rb1^{vi}$—Re1—$Rb1^{vii}$	180.000	$F1^{xii}$—Rb1—F1	62.7(2)
$F1^{i}$—Re1—Rb1	126.36(2)	$F1^{xiii}$—Rb1—F1	118.68(6)
$F1^{ii}$—Re1—Rb1	54.8(2)	$F1^{xiv}$—Rb1—F1	166.2(3)
$F1^{iii}$—Re1—Rb1	125.2(2)	$F1^{v}$—Rb1—F1	56.0(3)
$F1^{iv}$—Re1—Rb1	126.36(2)	$F1^{xv}$—Rb1—F1	118.68(5)
$F1^{v}$—Re1—Rb1	53.64(2)	$F1^{xvi}$—Rb1—F1	63.1(3)
F1—Re1—Rb1	53.64(2)	$F1^{xi}$—Rb1—$F1^{xvii}$	104.5(2)
$Rb1^{i}$—Re1—Rb1	180.000	$F1^{x}$—Rb1—$F1^{xvii}$	144.30(9)
$Rb1^{vi}$—Re1—Rb1	73.23(3)	$F1^{xii}$—Rb1—$F1^{xvii}$	144.30(9)
$Rb1^{vii}$—Re1—Rb1	106.77(3)	$F1^{xiii}$—Rb1—$F1^{xvii}$	52.0(2)
$F1^{i}$—Re1—$Rb1^{viii}$	126.36(2)	$F1^{xiv}$—Rb1—$F1^{xvii}$	52.0(2)
$F1^{ii}$—Re1—$Rb1^{viii}$	126.36(2)	$F1^{v}$—Rb1—$F1^{xvii}$	85.74(14)
$F1^{iii}$—Re1—$Rb1^{viii}$	53.64(2)	$F1^{xv}$—Rb1—$F1^{xvii}$	85.74(14)
$F1^{iv}$—Re1—$Rb1^{viii}$	54.8(2)	$F1^{xvi}$—Rb1—$F1^{xvii}$	114.25(9)
$F1^{v}$—Re1—$Rb1^{viii}$	125.2(2)	F1—Rb1—$F1^{xvii}$	114.25(9)
F1—Re1—$Rb1^{viii}$	53.64(2)	$F1^{xi}$—Rb1—$F1^{ii}$	144.30(9)
$Rb1^{i}$—Re1—$Rb1^{viii}$	73.23(3)	$F1^{x}$—Rb1—$F1^{ii}$	144.30(9)
$Rb1^{vi}$—Re1—$Rb1^{viii}$	73.23(3)	$F1^{xii}$—Rb1—$F1^{ii}$	104.5(2)
$Rb1^{vii}$—Re1—$Rb1^{viii}$	106.77(3)	$F1^{xiii}$—Rb1—$F1^{ii}$	85.74(14)
Rb1—Re1—$Rb1^{viii}$	106.77(3)	$F1^{xiv}$—Rb1—$F1^{ii}$	114.25(9)
$F1^{i}$—Re1—$Rb1^{ix}$	53.64(2)	$F1^{v}$—Rb1—$F1^{ii}$	52.0(2)

F1^{ii}—Re1—Rb1^{ix}	53.64(2)	F1^{xv}—Rb1—F1^{ii}	114.25(9)
F1^{iii}—Re1—Rb1^{ix}	126.36(2)	F1^{xvi}—Rb1—F1^{ii}	85.74(14)
F1^{iv}—Re1—Rb1^{ix}	125.2(2)	F1—Rb1—F1^{ii}	52.0(2)
F1^{v}—Re1—Rb1^{ix}	54.8(2)	F1^{xvii}—Rb1—F1^{ii}	62.2(2)
F1—Re1—Rb1^{ix}	126.36(2)	F1^{xi}—Rb1—F1^{vi}	144.30(9)
Rb1^{i}—Re1—Rb1^{ix}	106.77(3)	F1^{x}—Rb1—F1^{vi}	104.5(2)
Rb1^{vi}—Re1—Rb1^{ix}	106.77(3)	F1^{xii}—Rb1—F1^{vi}	144.30(9)
Rb1^{vii}—Re1—Rb1^{ix}	73.23(3)	F1^{xiii}—Rb1—F1^{vi}	114.25(9)
Rb1—Re1—Rb1^{ix}	73.23(3)	F1^{xiv}—Rb1—F1^{vi}	85.74(14)
Rb1^{viii}—Re1—Rb1^{ix}	180.000	F1^{v}—Rb1—F1^{vi}	114.25(9)
Re1—F1—Rb1^{x}	161.6(3)	F1^{xv}—Rb1—F1^{vi}	52.0(2)
Re1—F1—Rb1	95.10(14)	F1^{xvi}—Rb1—F1^{vi}	52.0(2)
Rb1^{x}—F1—Rb1	83.70(14)	F1—Rb1—F1^{vi}	85.74(14)
Re1—F1—Rb1^{viii}	95.10(14)	F1^{xvii}—Rb1—F1^{vi}	62.2(2)
Rb1^{x}—F1—Rb1^{viii}	83.70(14)	F1^{ii}—Rb1—F1^{vi}	62.2(2)

Symmetry codes: (i) -x, -y, -z; (ii) x-y, x, -z; (iii) -x+y, -x, z; (iv) y, -x+y, -z; (v) -y, x-y, z; (vi) 1-x, 1-y, -z; (vii) -1+x, -1+y, z; (viii) x, -1+y, z; (ix) -x, 1-y, -z; (x) 1-x, 1-y, 1-z; (xi) y, 1-x+y, 1-z; (xii) x-y, x, 1-z; (xiii) -x+y, 1-x, z; (xiv) x, 1+y, z; (xv) 1-y, 1+x-y, z; (xvi) 1-x+y, 1-x, z; (xvii) y, 1-x+y, -z.

Geometry.

Anisotropic displacement parameters [Å^2] for Cs_2ReF_6						
Atom	U_{11}	U_{22}	U_{33}	U_{12}	U_{13}	U_{23}
Re1	0.00483(16)	0.00483(16)	0.0032(2)	0.00241(8)	0.00000	0.00000
F1	0.0088(8)	0.0107(6)	0.0075(9)	0.0044(4)	-0.0023(6)	-0.0012(3)
Cs1	0.00661(16)	0.00661(16)	0.0052(2)	0.00331(8)	0.00000	0.00000

Geometric parameters, bond lengths [Å] and angles [°] for Cs_2ReF_6			
Re1—F1^{i}	1.9594(18)	F1—Cs1^{viii}	3.1655(6)
Re1—F1^{ii}	1.9594(18)	F1—Cs1^{vi}	3.224(2)

Re1—F1^{iii}	1.9594(18)	Cs1—F1^{xi}	3.0955(19)
Re1—F1^{iv}	1.9594(18)	Cs1—F1^{x}	3.0955(19)
Re1—F1^{v}	1.9594(18)	Cs1—F1^{xii}	3.0955(19)
Re1—F1	1.9594(18)	Cs1—F1^{xiii}	3.1655(6)
Re1—Cs1^{i}	3.9100(6)	Cs1—F1^{xiv}	3.1655(6)
Re1—Cs1^{vi}	3.9100(6)	Cs1—F1^{iii}	3.1655(6)
Re1—Cs1^{vii}	3.9100(6)	Cs1—F1^{xv}	3.1655(6)
Re1—Cs1	3.9100(6)	Cs1—F1^{xvi}	3.1655(6)
Re1—Cs1^{viii}	3.9100(6)	Cs1—F1^{xvii}	3.224(2)
Re1—Cs1^{ix}	3.9100(6)	Cs1—F1^{iv}	3.224(2)
F1—Cs1^{x}	3.0955(19)	Cs1—F1^{vi}	3.224(2)
F1—Cs1	3.1655(6)		
F1^{i}—Re1—F1^{ii}	93.14(7)	Cs1—F1—Cs1^{viii}	163.82(6)
F1^{i}—Re1—F1^{iii}	86.86(7)	Re1—F1—Cs1^{vi}	94.78(7)
F1^{ii}—Re1—F1^{iii}	180.00(4)	Cs1^{x}—F1—Cs1^{vi}	102.55(5)
F1^{i}—Re1—F1^{iv}	93.14(7)	Cs1—F1—Cs1^{vi}	94.07(3)
F1^{ii}—Re1—F1^{iv}	93.14(7)	Cs1^{viii}—F1—Cs1^{vi}	94.07(3)
F1^{iii}—Re1—F1^{iv}	86.86(7)	F1^{xi}—Cs1—F1^{x}	67.11(6)
F1^{i}—Re1—F1^{v}	86.86(7)	F1^{xi}—Cs1—F1^{xii}	67.11(6)
F1^{ii}—Re1—F1^{v}	86.86(7)	F1^{x}—Cs1—F1^{xii}	67.11(6)
F1^{iii}—Re1—F1^{v}	93.14(7)	F1^{xi}—Cs1—F1^{xiii}	62.38(6)
F1^{iv}—Re1—F1^{v}	180.00(8)	F1^{x}—Cs1—F1^{xiii}	129.122(17)
F1^{i}—Re1—F1	180.000	F1^{xii}—Cs1—F1^{xiii}	97.70(3)
F1^{ii}—Re1—F1	86.86(7)	F1^{xi}—Cs1—F1^{xiv}	62.38(6)
F1^{iii}—Re1—F1	93.14(7)	F1^{x}—Cs1—F1^{xiv}	97.70(3)
F1^{iv}—Re1—F1	86.86(7)	F1^{xii}—Cs1—F1^{xiv}	129.122(17)
F1^{v}—Re1—F1	93.14(7)	F1^{xiii}—Cs1—F1^{xiv}	53.43(7)
F1^{i}—Re1—Cs1^{i}	53.533(6)	F1^{xi}—Cs1—F1^{iii}	97.70(3)
F1^{ii}—Re1—Cs1^{i}	53.533(6)	F1^{x}—Cs1—F1^{iii}	129.122(17)
F1^{iii}—Re1—Cs1^{i}	126.467(6)	F1^{xii}—Cs1—F1^{iii}	62.38(6)
F1^{iv}—Re1—Cs1^{i}	124.74(6)	F1^{xiii}—Cs1—F1^{iii}	65.44(7)
F1^{v}—Re1—Cs1^{i}	55.26(6)	F1^{xiv}—Cs1—F1^{iii}	118.323(15)
F1—Re1—Cs1^{i}	126.467(6)	F1^{xi}—Cs1—F1^{xv}	97.70(3)
F1^{i}—Re1—Cs1^{vi}	124.74(6)	F1^{x}—Cs1—F1^{xv}	62.38(6)
F1^{ii}—Re1—Cs1^{vi}	53.533(6)	F1^{xii}—Cs1—F1^{xv}	129.122(17)
F1^{iii}—Re1—Cs1^{vi}	126.467(6)	F1^{xiii}—Cs1—F1^{xv}	118.323(14)
F1^{iv}—Re1—Cs1^{vi}	53.532(6)	F1^{xiv}—Cs1—F1^{xv}	65.44(7)
F1^{v}—Re1—Cs1^{vi}	126.468(6)	F1^{iii}—Cs1—F1^{xv}	163.82(6)
F1—Re1—Cs1^{vi}	55.26(6)	F1^{xi}—Cs1—F1	129.123(17)
Cs1^{i}—Re1—Cs1^{vi}	106.554(9)	F1^{x}—Cs1—F1	97.70(3)
F1^{i}—Re1—Cs1^{vii}	55.26(6)	F1^{xii}—Cs1—F1	62.38(6)
F1^{ii}—Re1—Cs1^{vii}	126.467(6)	F1^{xiii}—Cs1—F1	118.323(15)
F1^{iii}—Re1—Cs1^{vii}	53.533(6)	F1^{xiv}—Cs1—F1	163.82(6)
F1^{iv}—Re1—Cs1^{vii}	126.468(6)	F1^{iii}—Cs1—F1	53.43(7)
F1^{v}—Re1—Cs1^{vii}	53.532(6)	F1^{xv}—Cs1—F1	118.323(15)

F1—Re1—Cs1^{vii}	124.74(6)	F1^{xi}—Cs1—F1^{xvi}	129.122(17)
Cs1^{i}—Re1—Cs1^{vii}	73.446(9)	F1^{x}—Cs1—F1^{xvi}	62.38(6)
Cs1^{vi}—Re1—Cs1^{vii}	180.000	F1^{xii}—Cs1—F1^{xvi}	97.70(3)
F1^{i}—Re1—Cs1	126.467(6)	F1^{xiii}—Cs1—F1^{xvi}	163.82(6)
F1^{ii}—Re1—Cs1	126.467(6)	F1^{xiv}—Cs1—F1^{xvi}	118.323(15)
F1^{iii}—Re1—Cs1	53.533(6)	F1^{iii}—Cs1—F1^{xvi}	118.323(15)
F1^{iv}—Re1—Cs1	55.26(6)	F1^{xv}—Cs1—F1^{xvi}	53.43(7)
F1^{v}—Re1—Cs1	124.74(6)	F1—Cs1—F1^{xvi}	65.44(7)
F1—Re1—Cs1	53.533(6)	F1^{xi}—Cs1—F1^{xvii}	102.55(5)
Cs1^{i}—Re1—Cs1	180.000	F1^{x}—Cs1—F1^{xvii}	143.51(2)
Cs1^{vi}—Re1—Cs1	73.447(8)	F1^{xii}—Cs1—F1^{xvii}	143.51(2)
Cs1^{vii}—Re1—Cs1	106.553(8)	F1^{xiii}—Cs1—F1^{xvii}	49.86(5)
F1^{i}—Re1—Cs1^{viii}	126.467(6)	F1^{xiv}—Cs1—F1^{xvii}	49.86(5)
F1^{ii}—Re1—Cs1^{viii}	55.26(6)	F1^{iii}—Cs1—F1^{xvii}	85.93(3)
F1^{iii}—Re1—Cs1^{viii}	124.74(6)	F1^{xv}—Cs1—F1^{xvii}	85.93(3)
F1^{iv}—Re1—Cs1^{viii}	126.467(6)	F1—Cs1—F1^{xvii}	113.96(2)
F1^{v}—Re1—Cs1^{viii}	53.533(6)	F1^{xvi}—Cs1—F1^{xvii}	113.96(2)
F1—Re1—Cs1^{viii}	53.533(6)	F1^{xi}—Cs1—F1^{iv}	143.51(2)
Cs1^{i}—Re1—Cs1^{viii}	73.447(8)	F1^{x}—Cs1—F1^{iv}	143.51(2)
Cs1^{vi}—Re1—Cs1^{viii}	73.447(9)	F1^{xii}—Cs1—F1^{iv}	102.55(5)
Cs1^{vii}—Re1—Cs1^{viii}	106.553(9)	F1^{xiii}—Cs1—F1^{iv}	85.93(3)
Cs1—Re1—Cs1^{viii}	106.553(9)	F1^{xiv}—Cs1—F1^{iv}	113.96(2)
F1^{i}—Re1—Cs1^{ix}	53.533(6)	F1^{iii}—Cs1—F1^{iv}	49.86(5)
F1^{ii}—Re1—Cs1^{ix}	124.74(6)	F1^{xv}—Cs1—F1^{iv}	113.96(2)
F1^{iii}—Re1—Cs1^{ix}	55.26(6)	F1—Cs1—F1^{iv}	49.86(5)
F1^{iv}—Re1—Cs1^{ix}	53.533(6)	F1^{xvi}—Cs1—F1^{iv}	85.93(3)
F1^{v}—Re1—Cs1^{ix}	126.467(6)	F1^{xvii}—Cs1—F1^{iv}	64.10(5)
F1—Re1—Cs1^{ix}	126.467(6)	F1^{xi}—Cs1—F1^{vi}	143.51(2)
Cs1^{i}—Re1—Cs1^{ix}	106.553(8)	F1^{x}—Cs1—F1^{vi}	102.55(5)
Cs1^{vi}—Re1—Cs1^{ix}	106.553(9)	F1^{xii}—Cs1—F1^{vi}	143.51(2)
Cs1^{vii}—Re1—Cs1^{ix}	73.447(9)	F1^{xiii}—Cs1—F1^{vi}	113.96(2)
Cs1—Re1—Cs1^{ix}	73.447(9)	F1^{xiv}—Cs1—F1^{vi}	85.93(3)
Cs1^{viii}—Re1—Cs1^{ix}	180.000	F1^{iii}—Cs1—F1^{vi}	113.96(2)
Re1—F1—Cs1^{x}	162.67(9)	F1^{xv}—Cs1—F1^{vi}	49.86(5)
Re1—F1—Cs1	96.61(3)	F1—Cs1—F1^{vi}	85.93(3)
Cs1^{x}—F1—Cs1	82.30(3)	F1^{xvi}—Cs1—F1^{vi}	49.86(5)
Re1—F1—Cs1^{viii}	96.61(3)	F1^{xvii}—Cs1—F1^{vi}	64.10(5)
Cs1^{x}—F1—Cs1^{viii}	82.30(3)	F1^{iv}—Cs1—F1^{vi}	64.10(5)

Symmetry codes: (i) -x, -y, -z; (ii) y, -x+y, -z; (iii) -y, x-y, z; (iv) x-y, x, -z; (v) -x+y, -x, z; (vi) 1-x, 1-y, -z; (vii) -1+x, -1+y, z; (viii) x, -1+y, z; (ix) -x, 1-y, -z; (x) 1-x, 1-y, 1-z; (xi) y, 1-x+y, 1-z; (xii) x-y, x, 1-z; (xiii) -x+y, 1-x, z; (xiv) x, 1+y, z; (xv) 1-y, 1+x-y, z; (xvi) 1-x+y, 1-x, z; (xvii) y, 1-x+y, -z.
